# Covalent Organic Frameworks in Sample Preparation

**DOI:** 10.3390/molecules25143288

**Published:** 2020-07-20

**Authors:** Javier González-Sálamo, Gabriel Jiménez-Skrzypek, Cecilia Ortega-Zamora, Miguel Ángel González-Curbelo, Javier Hernández-Borges

**Affiliations:** 1 Departamento de Química, Unidad Departamental de Química Analítica, Facultad de Ciencias, Universidad de La Laguna (ULL), Avda. Astrofísico Fco. Sánchez, s/n°, 38206 San Cristóbal de La Laguna, Spain; alu0100945775@ull.edu.es (G.J.-S.); alu0100880090@ull.edu.es (C.O.-Z.); 2 Instituto Universitario de Enfermedades Tropicales y Salud Pública de Canarias, Universidad de La Laguna (ULL), Avda. Astrofísico Fco. Sánchez, s/n°, 38206 San Cristóbal de La Laguna, Spain; 3 Departamento de Ciencias Básicas, Facultad de Ingeniería, Universidad EAN, Calle 79 n° 11-45, 110221 Bogotá D.C., Colombia; magonzalez@universidadean.edu.co

**Keywords:** covalent organic frameworks, sample preparation, extraction, microextraction, sorbent

## Abstract

Covalent organic frameworks (COFs) can be classified as emerging porous crystalline polymers with extremely high porosity and surface area size, and good thermal stability. These properties have awakened the interests of many areas, opening new horizons of research and applications. In the Analytical Chemistry field, COFs have found an important application in sample preparation approaches since their inherent properties clearly match, in a good number of cases, with the ideal characteristics of any extraction or clean-up sorbent. The review article is meant to provide a detailed overview of the different COFs that have been used up to now for sample preparation (i.e., solid-phase extraction in its most relevant operational modes—conventional, dispersive, magnetic/solid-phase microextraction and stir-bar sorptive extraction); the extraction devices/formats in which they have been applied; and their performances and suitability for this task.

## 1. Introduction

The search for new extraction or clean-up sorbents with an applicability in Analytical Chemistry is a very active research area in sample preparation [1,2], the main characteristics being that the ideal sorbent should have a large specific surface area, high porosity and the ability to interact in a variety of ways with the target analytes. Selectivity and extraction efficiency are also key issues that should be properly evaluated in each case.

In this area of sample preparation sorbents research, relatively new (nano)porous materials called covalent organic frameworks (COFs), which could be considered as “organic zeolites” [3], were first synthesized in 2005 by Côté et al. [4], opening a new doorway for their application in different fields.

**Figure 1:**
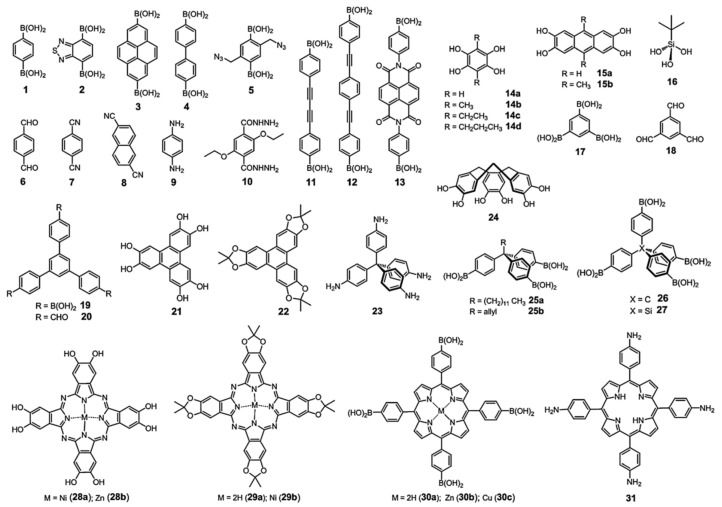
Some of the building units that have already been successfully used for the syntheses of covalent organic frameworks (COFs). Reprinted from Ding et al. [3] with permission of the Royal Society of Chemistry (RSC).

COFs are organic structures originated from the covalent bonding of light elements such as hydrogen, boron, carbon, nitrogen, oxygen, and silicon, in specific geometries. In fact, they are ordered crystalline organic polymeric structures with a very high porosity and a large specific surface areas, comparable in some cases to those of metal-organic frameworks (MOFs) [5,6]. COFs also show a density lower than those of MOFs as a result of their metal-free structures, though it is also possible to incorporate other atoms or functional moieties in them via post synthesis or bottom up strategies for different purposes [3,7], for example, to create electrocatalytic active sites [5]. It is also important to mention that, although COF structures are composed of light elements, in some cases, certain arrangements of the building blocks can give way to the incorporation of metal ions by means of coordination bonds, as can be seen in Figure 1. Like MOFs, COFs have molecular secondary structural units that can be tunable, such that different configurations are obtained within a periodic and well-defined architecture. Figure 1 shows some of the building units that have already been successfully applied for COFs’ syntheses. The symmetry, size and connectivity of the linkers predefine the geometry of the resulting framework. However, it should be remarked that the structural regularity of COFs is more difficult to achieve than it is for MOFs [3].

COFs can be classified according to the dimensions of the building units in the two-dimensional (2D) COFs, with the layers stacked via π-π interactions, and in three-dimensional (3D) COFs, when a 3D net is built. The last of them, firstly introduced in 2007 (COF-102 and COF-103) [8], though less common, and in general, with poorer porous homogeneity and lower crystallinities [6], have higher surface areas [9]. On the contrary, 2D-COFs are easier to synthesize as a result of their simpler structures.

**Figure 2:**
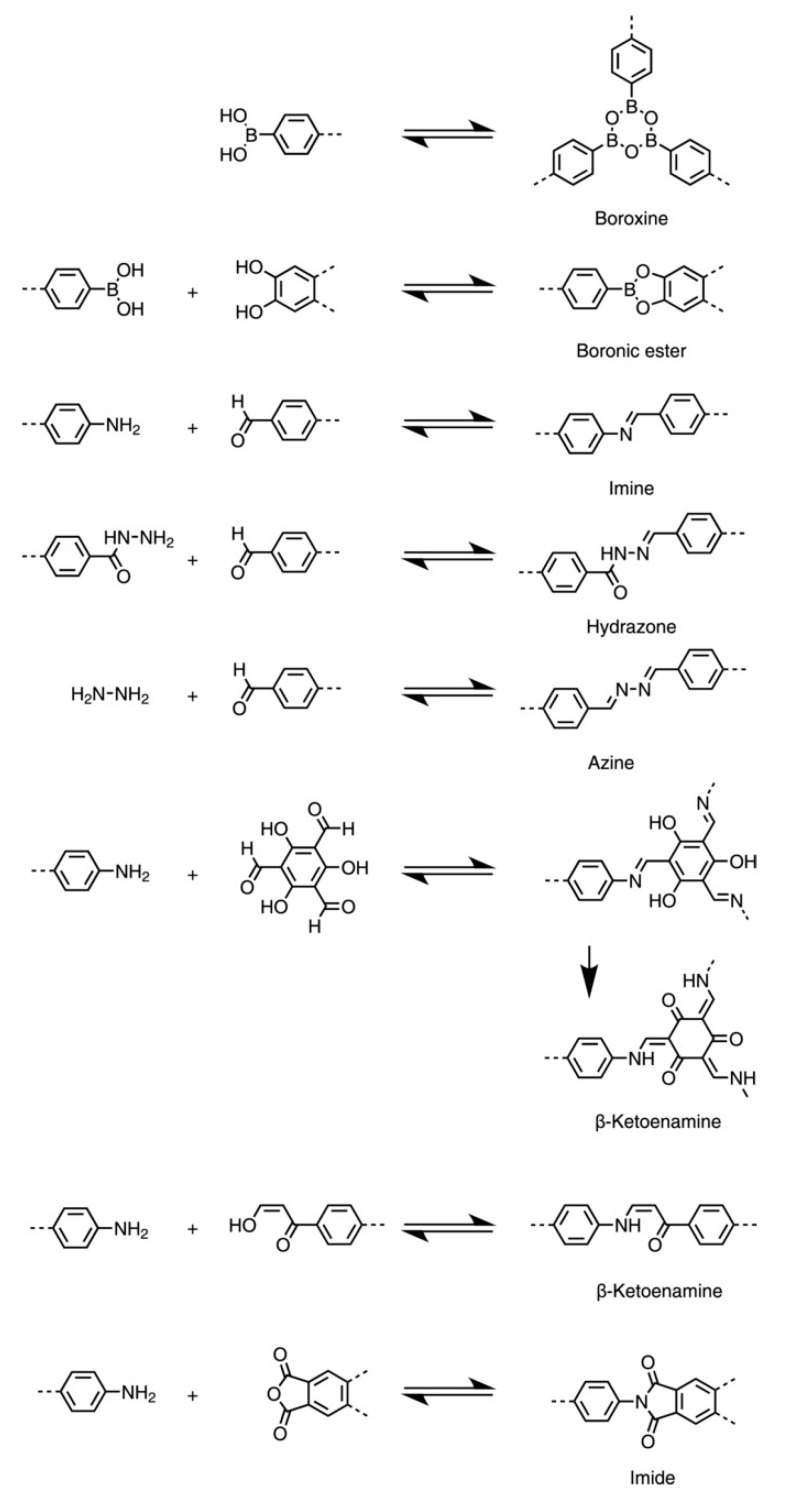
Widely applied condensation reactions for the formation of COFs. Reprinted from [10] with permission of Wiley.

In the first report of COFs [4], the authors proposed a general design strategy for the synthesis and crystallization of microporous and mesoporous crystalline COFs. The first of them that were synthesized, COF-1 [(C_3_H_2_BO)_6_·(C_9_H_12_)_1_] and COF-5 (C_9_H_4_BO_2_)-2D-COFs, were found to be stable up to 600 °C and were obtained with a simple “one-pot” procedure. Both COFs, which also showed low densities and specific surface areas (between 700 and 1600 m^2^/g) higher than those of well-known zeolites and porous silicates were produced by condensation reactions of boronic acids under mild conditions. Since then, a variety of different reactions have been successfully applied, which are compiled in Figure 2 [10].

A good number of other different methods that have been proposed up to now for COFs synthesis are based on solvothermal [3,4] or ionothermal [3,11] reactions, although other methods are currently being developed. As an example, recent research has also shown that some of them can be synthesized under room temperature and pressure [12,13,14], but such synthetic conditions still remain a challenge. More detailed information regarding the different synthesis procedures for obtaining COFs can be found in previous review articles in which this specific issue has been described [6,15,16,17].

COFs properties are related to the strength of the covalent linkages or the interlayer interaction—when 2D-COFs are considered. In this sense, though in general they have a good chemical and thermal stability (up to 600 °C [4,5]), boron-based COFs (boroxines and boronic esters)—the first synthesized—are sensitive towards hydrolysis [10]. However, those based on imine-linkage formed by the condensation between aldehydes and primary amines, and with azines, hydrazones and imides, are much more stable in organic solvents and water, a stability which is even higher for β-ketoenamines, which are formed from primary amines and 1,3,5-triformylphloroglucinol (Tp) [10]. Instead, boron-containing COFs have higher Brunauer-Emmett-Teller (BET) surface areas [3]. Such properties will clearly determine the applicability of COFs in a specific field.

COFs have been used for a wide variety of applications, among which their use in energy storage [17,18], gas storage and separation [3,19,20], catalysis [3,5,20] and photoelectronics [3,21] can be highlighted. In the analytical chemistry field, they have been used as parts of sensors [7,22]; in gas chromatography (GC) [12,23], in liquid chromatography (LC) [24,25] and in capillary electrochromatography [26,27] stationary phases; and mainly, as sorbents for analyte extraction and clean-up [28,29,30]. In fact, their use in sorbent-based extraction techniques, like other nanomaterials, has driven important advancements in the last few years, since their introduction has allowed overcoming some of the deficiencies of conventional sorbents, such as the poor extraction of polar compounds; low adsorption capacity and specificity for the analysis of trace or ultratrace analytes in complex samples; and the use of large volumes of solvents [31]. However, it is important to mention that their obtention follows complex synthetic routes in many cases, most of which cannot be developed at the industrial scale yet, making difficult their commercialization, contrary to most of conventional sorbents. In this sense, pristine and postmodified COFs have been directly used as solid-phase extraction (SPE) sorbents under its different modalities, i.e., conventional SPE, dispersive SPE (dSPE) or even magnetic dSPE (m-dSPE). COF composites or coated solid-phase microextraction (SPME) fibers or stir-bars have also been synthesized and applied with success to a variety of analytes and samples. This review article is aimed at providing an updated critical revision of the current state-of-the-art of the applications of COFs in sample preparation procedures, especially those related to their use as extraction sorbents. Some of the simplest COFs synthetic procedures have also been highlighted, especially when they have been combined with other nanomaterials to form the so-called hybrid COFs.

## 2. COFs as Sorbents in Solid-Phase Extraction

### 2.1. Conventional Solid-Phase Extraction

SPE is one of the sample preparation procedures mostly used nowadays. It is generally characterized by a lower consumption of solvents and easy operation compared to liquid-liquid extraction (LLE) and by high enrichment factors and flexibility in the use of sorbents [32,33]. Conventional SPE uses closed cartridges or columns packed with the stationary solid-phase and it generally consists of four stages, i.e., conditioning, retention, drying and elution, although sometimes an additional washing step is performed before drying/elution in order to eliminate as many interferences as possible [34], especially in those cases in which complex matrices are analyzed.

One of the most active research areas of SPE is the synthesis of new extraction sorbents able to extract the target analytes selectively and quantitatively. An ideal SPE sorbent should have a large surface area; good physical and chemical stability; and high sorption capability and rapid mass transfer capacity [35]. COFs clearly meet such requirements, and therefore, they have been widely used as sorbents in conventional/classical SPE, as can be seen in Table 1.

The variety of analytes determined by SPE using COFs as sorbents, is wide. Among those shown in Table 1, there can be found pesticides such as benzoylurea insecticides [36,37] pyrethroids [38] and carboxylic acid pesticides [39]. All of them have an endocrine disrupting activity and some of them are potentially carcinogenic and teratogenic, such as certain phenolic and bisphenolic compounds which have also been extracted using COF-SPE [40]. Moreover, sulphonamides (SAs) [41,42] and inorganic trace ions [43] have also been determined. Other target analytes have been biogenic amines [44], as indicators of the freshness and hygiene of food during storage, especially of meat products, and water disinfection by-products containing chlorine [45], which also have carcinogenic, teratogenic and mutagenic effects.

The types of samples analyzed have been very diverse, as also shown in Table 1. They include fruits [37,38], juices [36,37], vegetables [36,37,38], plant extracts used in traditional Chinese medicine [38], environmental samples and drinking water [37,39,41,43,45], milk [40,41,43], carbonated beverages [40] and meat [41,42,44].

Liquid samples, such as water [37,39,41,43,45] and other beverages [40], have been directly extracted with COF sorbents after previous filtration (and degasification for the carbonated ones), whereas solid or semi-solid samples have been, in some cases, first extracted using an organic solvent. In the case of a milk sample, for example, a previous deproteinization step with the help of an acidifying agent for protein precipitation and a subsequent centrifugation is required, and a filtration for the following SPE procedure [40,41]. On the other hand, other samples such as meat [41,42,44] have been first minced and a certain solvent, such as acetonitrile (ACN), or even an acid, such as trichloroacetic acid, has been added before it was stirred and centrifuged. In addition, some works have also added n-hexane to previously remove fats [44]. Concerning the pre-treatment of fruit and vegetable samples, it frequently involves their crushing into small pieces, homogenization, centrifugation and filtering of the supernatant [36,37,38]. The COFs that have been used for such purposes can be classified into three groups in terms of composition: conventional or as synthesized COFs [36,37,40,44,45], functionalized COFs [39,43] and hybrid COFs, which result from the combination with other materials—for example, MOFs [41] or polymers [38,42], among others. COFs belonging to the first group (COFs directly used in SPE) are synthesized following a series of stages from their building blocks. Most of these COFs have in common the use of Tp as a building block, which has been prepared at the laboratory in a good number of works prior to the final synthesis of the COF following the same synthesis procedure: mixing hexamethylenetetramine, phloroglucinol and trifluoroacetic acid under a N_2_ atmosphere and heating the mixture in a water bath at 80 °C for 3 h [36,37,44]. The next step before the final COF was obtained was similar for most works dealing with their use in SPE, but the amounts of reagents varied. Said procedure consisted of the addition of p-toluene sulfonic acid to a mortar together with the other building blocks (4,4′-azodianiline (Azo), 2,6-diaminoanthraquinone (DA), 2-nitro-p-phenylenediamine (Pa-NO_2_), melamine (MA) and 4,4′-diaminobiphenyl (BD), respectively), and the grounding of the mixture to a powder. Then, Tp was added, and the grinding continued until a dark red color appeared. A certain volume of water was added, and a paste was formed, which was heated in an oven (a deep reddish-brown powder was obtained in most cases) [36,37,40,44,45].

Regarding functionalized COFs, the obtaining of the COF called CTpBD (where C refers precisely to the carboxylic acid groups introduced in the Tp building block) followed a procedure very similar to that of the previously mentioned works where all of them have the Tp building block in common, but with a difference in that the Tp had to be first functionalized with diglycolic anhydride to bind the carboxylic acid groups. Then, the obtained COOH-Tp was mixed with the BD and refluxed in argon atmosphere [43].

Ji et al. [39] have also synthesized a functionalized COF which they called NH_2_@COF. For this purpose, 1,3,5-*tris*(4-aminophenyl)benzene (TAPB) and 2,5-divinylterephthalaldehyde (Dva) were mixed with 1,4-dioxane, n-butanol and an acetic acid aqueous solution to obtain the vinyl COF. The latter was combined with 4-aminobenzenethiol, azobisisobutyronitrile (AIBN) and trifluorotoluene to obtain the NH_2_@COF. The surface area and the pore volume of the COF were studied, and it was determined that it had a high porosity which made this COF suitable for packing SPE cartridges. This novel amino-modified COF was used for the extraction of six carboxylic acid pesticides from water samples (river, lake, ground and tap water)—it showed high selectivity for these analytes—and subsequent analysis by high-performance liquid chromatography (HPLC) coupled to a diode array detector (DAD). Additionally, a comparison between the results obtained using this functionalized COF, a non-functionalized COF and other commercial adsorbents such as C_18_, silica, phenyl-silica, and SAX was made. NH_2_@COF achieved the highest extraction efficiency, with recovery values between 89.6% and 102.4% and relative standard deviation (RSD) values in the range 0.03–7.10%.

**Figure 3:**
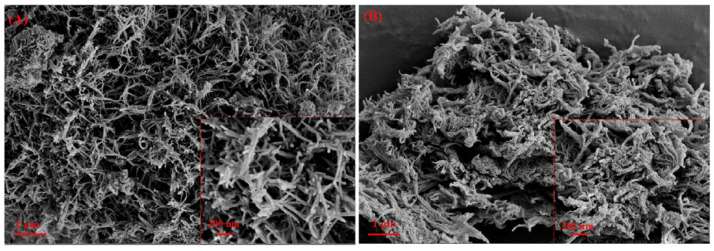
SEM images of MICOFs-7 (**A**) and NICOFs-7 (**B**). Reprinted from [38] with permission of Elsevier.

Concerning hybrid COFs, Ji and co-workers [38] synthesized for the first time, molecularly imprinted COFs (MICOFs) in order to take advantage of the high selectivity, chemical stability, relatively low cost and ease of preparation of molecularly imprinted polymers (MIPs) [46]. For this purpose, TAPB and Tp were mixed at room temperature in the presence of fenvalerate (the template) and Sc(OTf)_3_ as the catalyst. The results were compared with those obtained with the non-imprinted COFs (NICOFs). In addition, it was determined that the affinity and efficiency of the MICOFs varied depending on the amount of template used in the preparation. The characterization of the MICOFs was performed using scanning electron microscopy (SEM) (see Figure 3) and Fourier transform infrared spectroscopy (FTIR), and their permanent porosities were defined using the BET method. MICOFs were observed to have a 3D structure which consisted of a large number of nanofibers that had aggregates of different sizes and morphologies. These imine-linked MICOFs were applied to the extraction of four cyanopyrethroids from vegetables, fruits and traditional Chinese medicines. The developed method was simple and sensitive (the limits of detection—LODs—in the range 0.011–0.018 μg/kg) and provided high recovery percentages for the target compounds (in the range 94.3–102.1%) with high precision (RSDs between 3.1% and 5.9%).

It is also worth mentioning the article of Chen et al. [41], in which the synthesized sorbent was a MOF@COF hybrid. Since COFs are analogous to MOFs but with some advantages over them [47], these researchers attempted to integrate a MOF (NH_2_-MIL-68) and a COF (*tris*(4-formylphenyl)amine (TFPA) and *tris*(4-aminophenyl)amine (TAPA)) to form a new type of hybrid material, with the aim of combining the advantages of both materials. In this case, the procedure followed was the synthesis of the yellow MOF (NH_2_-MIL-68), which was then functionalized with TFPA to form NH_2_-MIL-68 (CHO). Finally, the brown powder of the hybrid material MOF@COF was obtained by reacting this functionalized MOF together with TFPA and TAPA for a certain time and using 4:1 (*v*/*v*) o-dichlorobenzene:ethanol (EtOH) as solvent. The optimized method was used for the extraction of SAs from tap water, meat and milk samples, and recovery values between 68.9% and 103.8% were obtained with high precision (RSDs in the range 2.9–6.6%). Furthermore, a study was carried out to verify that this hybrid COF had better properties and yields than the separate MOF and COF in the extraction of SAs. It was obtained that the signal intensity of the adsorbed analytes was higher for the COF than for the MOF; therefore, the importance of the COF layer was deduced. However, it was much higher when MOF@COF was used, due to its high porosity and surface area, and the multiple specific interactions between the hybrid COF and the target compounds. However, this method also had some disadvantages, such as high packed column pressure and the fact that high volume samples could not be analyzed.

**Figure 4:**
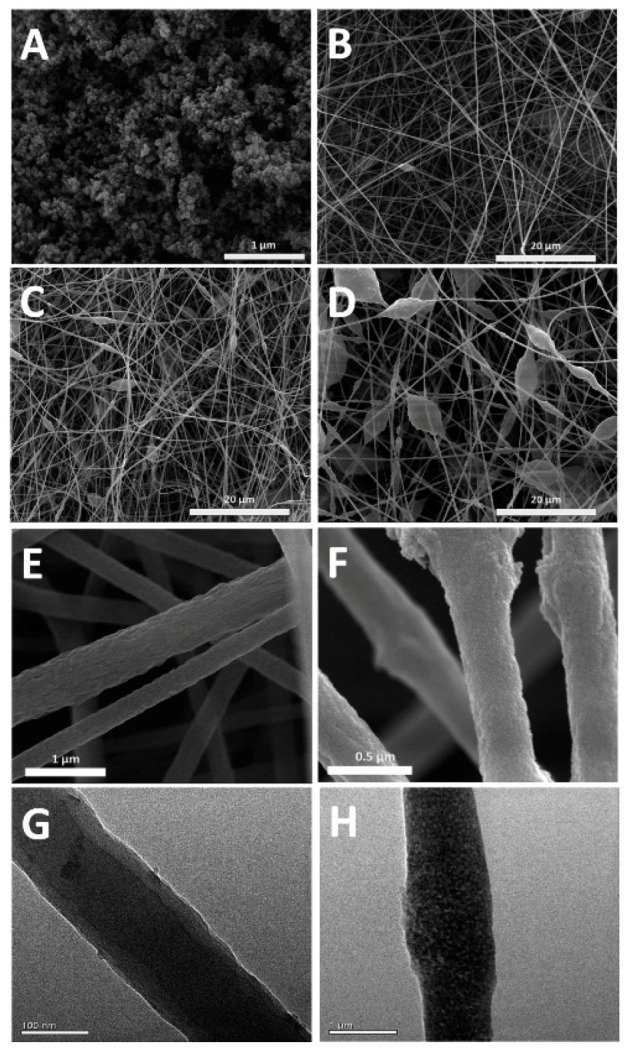
SEM images of SNW-1 (**A**), SNW-1@PAN 0.05 (**B** and **F**), SNW-1@PAN 0.1 (**C**), SNW-1@PAN 0.2 (**D**) and PAN nanofiber (**E**); TEM images of PAN (**G**) and SNW-1@PAN 0.05 (**H**). Reprinted from [42] with permission of Elsevier.

Although it is not a conventional SPE approach, it should be highlighted that COFs have been used as sorbents for pipette tip SPE (PT-SPE) on a few occasions [41,42], probably because their direct packaging into pipette tips could cause leakage problems and high backpressure in such a tip as a consequence of the micron and submicron sizes of the COF particles. To minimize these drawbacks, Yan et al. [42] synthesized and applied for the first time a COF (SNW-1) incorporated polyacrylonitrile (PAN) electrospun (SNW-1@PAN) nanofiber as a sorbent in PT-SPE through a co-electrospinning method for the analysis of five SAs in pork and chicken, whose SEM image is shown in Figure 4C. In this way, the leakage and high backpressure produced by packaging the COF straight away into the PT-SPE device are reduced due to the 3D networks of the electrospun nanofiber. Moreover, SNW-1 is an off-white powder that presents the typical advantages of a COF (3D framework with high specific surface area, good chemical and thermal stability, among other things) formed by C-N bonds between two monomers of MA and terephthalaldehyde (TA) [48].

In most of the works shown in Table 1, a study under optimal extraction conditions was carried out in order to evaluate the stability and reusability of each COF sorbent. It was frequently found that they could be reutilized between 20 and 50 times depending on the COF, without significant losses in the recovery of the analytes and without significant carry over. Concerning the hybrid material NH_2_-MIL-68@COF, it was found that it could be reused up for 100 times for the extraction of six SAs from environmental water, milk and meat samples [41], while SNW-1@PAN could only be reused for five cycles for the extraction of five SAs from meat extracts [42].

All the COFs-SPE sorbents described above were used under similar extraction conditions. Concerning the amount of sorbent, 20, 25 or 100 mg was used in most cases, except in those cases wherein PT-SPE was used, in which the amount of sorbent was lower, 8 [41] or 12.5 mg [42]. The conditioning step is generally performed with ACN, acetone, methanol (MeOH) and distilled water, although in some works other solvents or solutions were used, such as HNO_3_ (0.5 M) and NH_4_Ac (0.1 M) [43], NH_3_:MeOH 8:92 (*v*/*v*) [39] and EtOH and n-hexane [38]. The volume of the sample/extract varied between 0.5 and 200 mL, and some of them even required pH adjustments [39,41,43]. A washing stage has also been found to be necessary in some cases [36,37,38,42,44,45], using ACN, water, acetone, MeOH and n-butanol, or mixtures of them, with volumes between 1 and 10 mL. Concerning the elution step, it has been performed in many cases with ACN, although others such as HNO_3_ (0.7 M) [43], dichloromethane (DCM) [45] and acetic acid solutions [38,40], and mixtures, such as NH_3_-MeOH [39,42], have also been used. As can be seen, the use of COFs as sorbents frequently has a good compatibility with many organic solvents.

### 2.2. Dispersive Solid-Phase Extraction

SPE has become one of the main extraction techniques in sample preparation since its introduction in 1972 [49] due to its well-known advantages with respect to other solvent-based procedures. However, some drawbacks, such as the need for conditioning and sample loading steps, resulting in long extraction times; difficulties in performing more than one extraction at a time; and carry-over problems derived from the reusability of the sorbents or cartridges blocking caused by particles and microorganisms from the sample matrix, made necessary the introduction of sorbent-based extraction alternatives in order to solve those problems/drawbacks [50]. In this sense, multiple modifications of the original procedure have been proposed, including several miniaturized versions following the same concept, such as PT-SPE or spin column SPE, among others, as previously indicated [51]. However, the dSPE version meant a revolution for this technique in terms of simplification and time savings, since the direct dispersion of the sorbent into the sample matrix allowed one to avoid conditioning and sample loading steps, considered as the bottlenecks of conventional SPE. At the same time, dSPE allows the analysis of complex matrices, such as environmental and food samples, without classical blocking problems [50]. Although dSPE was initially introduced as a clean-up procedure as part of the QuEChERS (quick, easy, cheap, effective, rugged and safe) method [52], it has shown a good performance when it is applied with extraction purposes. In this case, after sorbent dispersion, it is separated from the sample by centrifugation or by filtration/retention in, for example, an empty column using an appropriate frit. Then, the sorbent can be washed if necessary and dried, and the analytes are desorbed or eluted with a suitable solvent.

Like any other SPE procedure, the sorbent selection is also crucial in dSPE, in which nanomaterials have emerged as one of the most interesting alternatives due to their high surface-to-volume ratio, their good chemical and physical properties and their occasional ability to tolerate modifications, among other things [53]. In this sense, despite other nanomaterials such as carbon nanotubes (CNTs) and MOFs being highly used as sorbents, in recent years the applicability of COFs has also been evaluated, although in a reduced number of works, as is shown in Table 2. As it can be seen in the table, COFs have been applied to the extractions of compounds various in nature, such as non-steroidal anti-inflammatory drugs (NSAIDs) [54,55], nitroaromatic compounds (NACs) [56], fluorochemicals [57], ultraviolet (UV) filters [58], pyrethroids [59] and N-nitrosamines [60] by the direct dispersion of the COF into the sample matrix after filtration. In most cases, the studied matrix has been water (different origins and types), except in one case, in which a migration study from food packaging materials was carried out [58].

**Figure 5:**
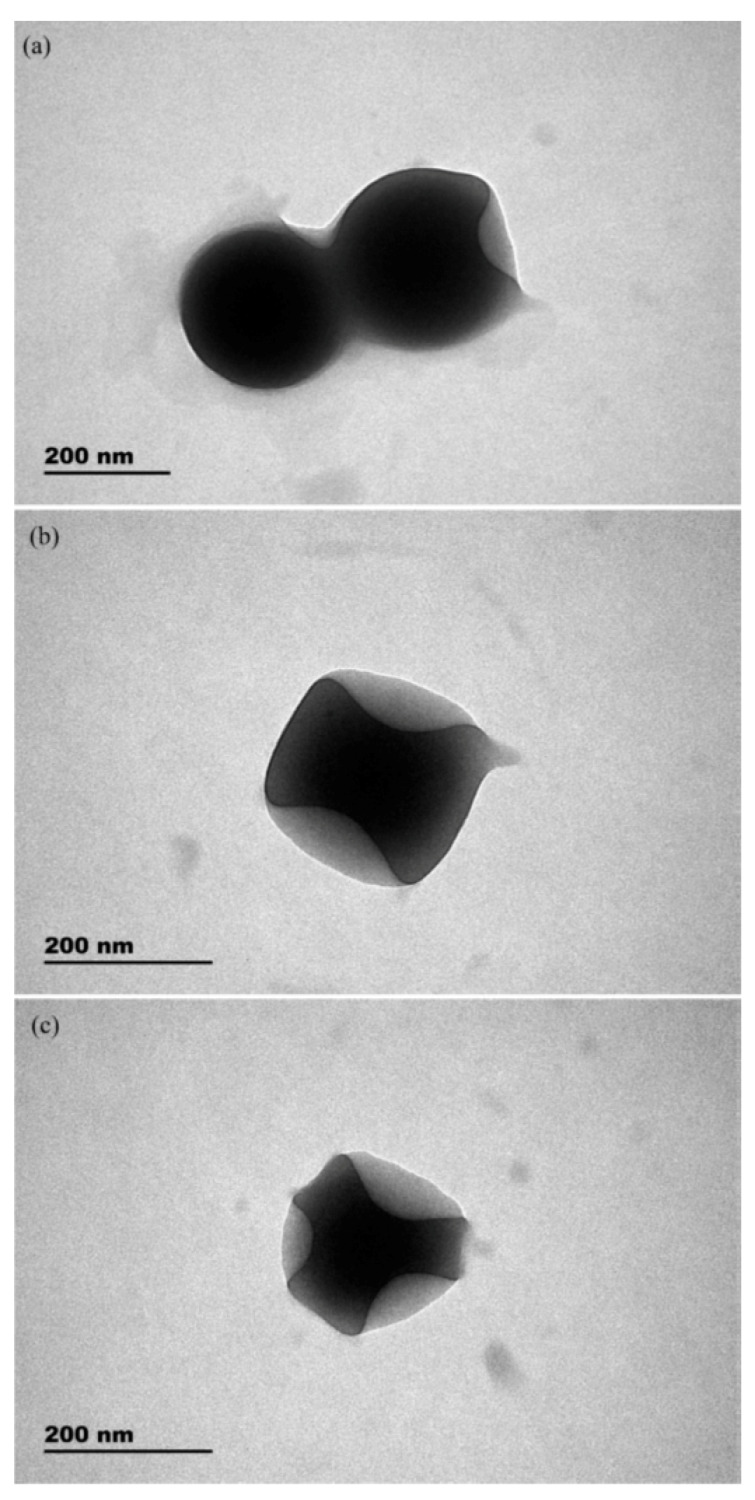
Transmission electron microscopy (TEM) images of nano-titania functionalized COFs, i.e., SSTF-COFs (**a**), DSTF- COFs (**b**) and CSTF-COFs (**c**), at different solvothermal reaction stages of 24, 48 and 72 h, respectively. Reprinted from [60] with permission of Elsevier.

As it has already been mentioned, in sorbent-based extraction techniques, the composition of the sorbent is very important in order to provide not only a high extraction capacity, but also a good selectivity. Following this principle, the selection of the building blocks used for the syntheses of COFs plays a very important role, so the number of building block combinations almost matches the family of analytes studied. In this format, COFs composed of 1,3,5-triformylbenzene (TFB)/BD [54,55], 2,3,5,6-tetrafluoroterephthalaldehyde (TFA)/TAPB [56], TFB/*p*-phenylenediamine (Pa) [57], Tp/Pa [58,59] and TFB/4,4′-diamino-p-terphenyl (DATP) [60] were used as synthesized [56,57], functionalized [58,60] or in combination with another material, such as silica or attapulgite [54,55,59]. Among the different COFs and combinations, it is worthy to mention the work of Zhang and co-workers [60], in which the extraction performances of three nano-titania functionalized COFs taken at different solvothermal reaction stages were evaluated. In this case, three batches of COFs were prepared by dissolving TFB and DATP in a mixture of mesitylene, 1,4-dioxane and aqueous acetic acid, and then submitted to ultrasound for 30 min to be finally introduced in a polytetrafluoroethylene (PTFE)-lined stainless-steel autoclave. The batches were reacted at 90 °C for 24, 48 and 72 h, which allowed obtaining three different structural morphologies, called the single roll-up shaped nano-titania functionalized COF (SSTF-COF), the double roll-up shaped nano-titania functionalized COF (DSTF-COF) and the clover-shaped nano-titania functionalized COF (CSTF-COF), respectively, as shown in Figure 5. After their cooling, the three compounds were separated by centrifugation, washed and vacuum dried. Then, every batch was functionalized with tetrabutyl titanate in dimethylformamide at 200 °C for 24 h in a PTFE-lined stainless-steel autoclave and roasted at 300 °C for 2 h. Finally, the functionalized COFs were washed and vacuum dried. The three modified COFs were evaluated as sorbents for the extraction of *N*-nitrosamines from drinking water. Although in general terms the three shapes provided good extraction capacity, with recovery values in the range 71.2–114.2%, CSTF-COF recovery was found to be better for all the analytes (85.1–98.5%), with RSD values much lower compared to SSTF-COF and DSTF-COF. Besides, the performance of the CSTF-COF as a dSPE sorbent was compared with Oasis^®^ HLB SPE cartridges, showing better extraction efficiency, precision and LODs, all while constituting a simpler and faster alternative.

Regarding dSPE procedures, sorbents have been directly dispersed into the samples using different mechanisms, including sonication [55,56], orbital shaking [56], vibration [57], manual shaking [58] and vortexing [59,60], but in all cases, the sorbent was separated by centrifugation prior to analyte desorption with a suitable organic solvent, which varied depending on the nature of the target analyte. They included individual solvents, such as ACN [56,58,59] or MeOH [60], combinations of both of them [57] or organic solvents containing bases [55]. From a procedural point of view, it is worth mentioning the work of Li and co-workers [54], in which the authors synthesized COF-functionalized poly (styrene-divinyl benzene-glycidylmethacrylate) (PS-GMA) particles by dispersing the PS-GMA particles in the hydrothermal reaction media for the synthesis of the COF. After being washed with MeOH and tetrahydrofuran, the sorbent was accurately weighed into a 10-mL syringe with a filter holder in order to prevent sorbent loses. Then, the sample was aspired into the syringe by pulling the plunger and dispersing the PS-GMA@COF particles; and after 1 min the solution was dispensed by pushing the plunger—this procedure repeated thrice. The excess of water was removed by pulling and pushing the plunger several times, and 0.5 mL of MeOH was loaded into the syringe, kept inside for 2 min and dispensed back in the same recipient; the desorption procedure was repeated thrice. Finally, the eluent was collected and 5 µL was injected in the ultra-high-performance liquid chromatography (UHPLC) system coupled to an UV detector. This in-syringe dSPE procedure showed high extraction capacity for all the NSAIDs in all tap water, river water and hospital waste-water samples analyzed, with recovery values in the range 84.3–99.6% and RSD values between 0.2% and 9.4%. In addition, LODs as low as 0.13–0.82 µg/L were obtained.

After dSPE, analytes have generally been separated by UHPLC [54,60] or HPLC [55,56,58,59] prior to their detection using UV [54,55,58], DAD [56,59] or mass spectrometry (MS) [60] detectors. The good extraction capacity shown by COFs in combination with these techniques allowed obtaining LODs in the order of low µg/L or even ng/L or ng/kg in most cases. Notwithstanding the good sensitivity achieved by these techniques, surface-assisted laser desorption/ionization-mass spectrometry (SALDI-MS) was also successfully applied in a very interesting application carried out by Wang et al. [57]. In their work, authors used the so called LZU1 COF (composed of TFB and Pa), synthesized following a solvothermal method, as both sorbent in dSPE and matrix of SALDI-time of flight (TOF) MS for the analysis of six fluorochemicals in tap and waste-water samples. The dSPE procedure consisted of the direct dispersion of the LZU1 COF in water sample under constant vibration for 90 min. After that time, the sorbent was separated by centrifugation and the supernatant was discarded to desorb the analytes with a mixture MeOH:ACN 1:1 (*v*/*v*). Finally, one microliter of it was deposited on the MALDI target for SALDI-TOF MS analysis. The use of LZU1 COF as the matrix when the MS was operated in the negative ion mode not only provided an important increase of the surface area for the absorption and transfer of the laser energy, but also considerably improved the reproducibility of the technique.

As it has been previously mentioned, the introduction of nanomaterials into these kinds of extraction procedures posed an important advance, since their extraordinary high surface-to-volume ratios allowed for a reduction of sorbent amount while maintaining a good extraction performance. In this sense, the introduction of magnetic nanoparticles (m-NPs), both as sorbent and as part of it, constituted a significant simplification for the dSPE procedures used until then. In the cases in which m-NPs are used, the procedure is called m-dSPE, and although the first step of the procedure is developed as in a dSPE, the sorbent isolation is carried out by using an external magnetic field. This allows for retaining the sorbent into the extraction recipient and removing the sample matrix by decantation, while avoiding the typical centrifugation or filtration/retention step and reducing the extraction time considerably [50,92]. Then, the sorbent is dispersed again in a suitable solvent for desorbing the analytes, and after a certain time, it is isolated with the magnet to recover the solvent containing the analytes by decantation [50,92].

Despite the inherent advantages of using m-NPs in dSPE procedures, this kind of nanomaterial does not normally show as good an extraction capacity and selectivity as others, so they are typically combined with other materials or even nanomaterials following different strategies in order to improve extraction performance. In this sense, the combination of m-NPs with COFs as sorbents in m-dSPE procedures constitutes the main application of COFs as sorbents by far. As can be seen in Table 2, magnetic COFs have been applied for the extraction of a wide variety of analytes, including polycyclic aromatic hydrocarbons (PAHs) [61,63,66,71], phthalic acid esters (PAEs) [64,69,78,86], perfluorinated compounds [65,91], hydroxylated polychlorinated biphenyls (PCBs) [68], phenolic compounds [67,72,76], estrogenic compounds [70,72], fluoroquinolones [73,88], dyes [74], antibiotics [75,80], heterocyclic aromatic amines (HAAs) [77,83], hydroxylated PAHs [79] and pesticides [81,82,84,90] originating from, among other places, environmental samples (water [61,63,65,66,80,82] and soils [63]), biological sources (rat plasma [62], human serum [67,68], human plasma [69,73], and human urine [70,77,79,87]) or food samples (beverages [76,84,85,86], meat [66,71,72,73,75,80,83,88], fish [66,71], coffee [66], oil [71], shrimp [72,80], milk [73,78,80,91], vegetables [81,84,89], fruit [84,85,89,90], and alcoholic beverages [86]), plastics [64] and textile samples [74]. They have shown very good performances in all cases.

Leaving apart the specific syntheses of COFs, multiples strategies have been used to combine COFs and m-NPs, resulting in different structures and morphologies depending on the building blocks and the way in which both nanomaterials have been combined. Regarding the building blocks used for the syntheses of magnetic COFs, in a similar way as previously mentioned for dSPE, a wide variety of building blocks can be found, since their selection depends in a certain way on the analytes to be extracted. However, what is clear is that the couple Tp and BD has been the preferred building block combination in m-dSPE procedures by far [66,72,75,77,85,86,88]. Although this has been the most usual, up to seventeen different combinations have been used with a total of twenty-three different building blocks. It is also important to mention that, in some cases, the COF has been composed of only one building block; e.g., benzene-1,4-diboronic acid (BDBA) [62] and 1,4-dicyanobenzene (DCB) [64,65,84].

**Figure 6:**
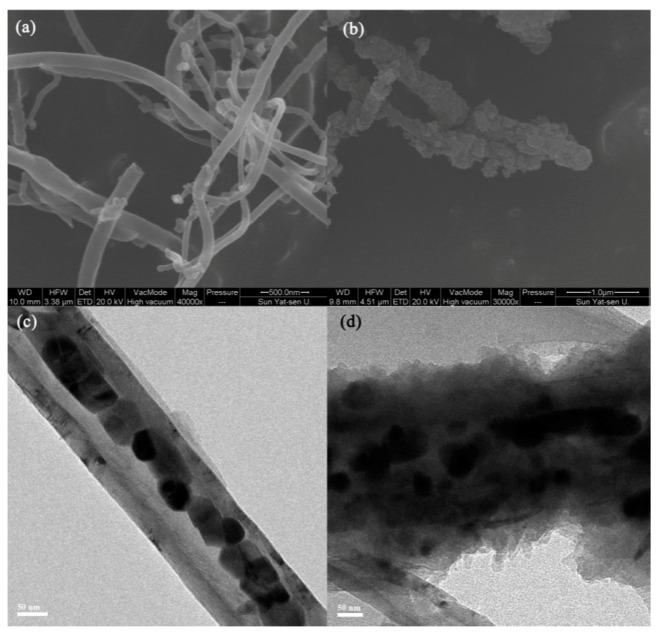
The SEM (**a**,**b**) and TEM (**c**,**d**) spectra of CNTs (**a**), magnetic CNT (**c**) and CoFe_2_O_4_@CNT@COF (**b**,**d**). Reprinted from [83] with permission of Elsevier.

Regarding the magnetization step, Fe_3_O_4_ m-NPs have been the most used without any doubt [61,62,63,66,67,68,69,70,71,72,73,74,75,76,77,78,79,80,81,82,85,86,87,88,89,90,91], although Ni [64,84], Fe_2_O_3_ [65] and CoFe_2_O_4_ [83] have also been applied. As previously mentioned, a wide variety of synthetic routes have been used to obtain different magnetic COFs, it being almost possible to find a procedure for each application. In this sense, one of the most common strategies consists of the in-situ growth of the COF around the m-NPs [61,62,68,69,70,71,72,73,74,75,76,77,79,80,81,84,86,89,90,91], normally resulting in a core-shell structure. In these cases, the m-NPs are generally suspended in a solution containing the building blocks. This step can be followed by simple sonication [62,67,69,70,76] and reaction at a certain temperature [73,74,75], or the solution can be introduced in a glass ampule, frozen with liquid N_2_, submitted to vacuum and sealed, to be finally heated at 120 °C for 3 days [71,72,80,89], although some particular variations have been introduced in each case. In fact, a typical modification of the last methodology consists of a previous functionalization of the m-NPs with one of the building blocks, and then, the functionalized m-NPs are dispersed with both building blocks to continue with the synthetic process [61,68,77,79,81,84,86,90,91]. However, there are some other alternatives that imply the reduction of metallic salts in the presence of COF, thereby obtaining a composite of COF with m-NPs distributed in its structure [64,65,82,84]. It is also important to mention that in many cases, the modification of the surface of m-NPs before their combination with COFs has been necessary in order to provide a suitable chemical surface to maximize the interaction between both nanomaterials. For this purpose, functionalization with NH_2_ groups [68,75,80,87,90,91], SiO_2_ [68,77,79,81,85,87,91] or polymers (polyethyleneimine [62,82], polydopamine (PDA) [62,69,78] or polyethylene glycol [82]) has been used. Finally, it should be highlighted that, despite the already proven good features of COFs as sorbents when they are used alone or with m-NPs, in certain applications, they have also been combined with carbon-based nanomaterials in order to take advantage of their π-π systems [78,83]. As an example, it is worth mentioning the work of Liang et al. [83]. In this case, the authors synthesized a cyclotricatechylene (CTC)-based COF around magnetic CNTs and used them for the extraction of nine HAAs from fried chicken and roast beef samples. The first synthetic step consisted of the hydroxylation of CNTs and its magnetization by dispersing them in an ethanolic solution containing Fe(NO_3_)_3_ and Co(NO_3_)_2_ in a 2:1 molar ratio. After sonication, the mixture was dried at 60 °C; then maintained at 100 °C for 2 h; and finally heated at 550 °C for 2 h to obtain CoFe_2_O_4_-filled CNTs as shown in Figure 6 (in particular, in Figure 6c). Once magnetic CNTs were synthesized, the CoFe_2_O_4_@CNT@COF NPs were obtained via a photochemical process. To this, magnetic CNTs, BDBA and CTC were introduced into a quartz bottle and sealed and degassed with N_2_, thereby producing an inert atmosphere. A 1,4-dioxane:mesitylene 1:1 (*v*/*v*) solution and 3-aminopropyltriethoxysilane (APTES) were added, and the mixture was sonicated for 1 h and irradiated with UV light for 48 h. After washing and drying the sorbent, it was ready to be used. This composite showed improved stability and a good extraction capacity which, in combination with UHPLC coupled to tandem mass spectrometry (MS/MS) allowed obtaining LODs in the range of 0.0058–0.025 µg/kg for all the analytes and matrices.

Regarding the extraction procedure, the very varied nature of the analyzed samples has resulted in the need to include different stages prior to the application of the different magnetic sorbents. Thus, it is possible to find anything from simple filtering procedures for water samples [61,63,65,66], dilutions for biological samples (among others) [67,70] and blending procedures for fruit and vegetable samples [81,85,89,90], to elaborate deproteinization/digestion procedures for milk and biological samples [62,68,69,73,78,80,83,88] and hydrolysis for biological, meat and fish samples [66,71,77,79], among other specific sample pre-treatment procedures. In this sense, it is important to mention that, in some cases, a previous extraction with organic solvents is needed after applying some of the previously mentioned sample pre-treatments. As an example, Lin and co-workers [90] applied Fe_3_O_4_@N-[3-(trimethoxysilyl)propyl]ethylenediamine (PSA)@COF as the sorbent for the extraction of 20 organophosphorus pesticides from different fruit samples, including watermelon, peach and orange samples. In this case, every fruit sample was homogenized, and 10 g was accurately weighted before being submitted to the classical first step of the QuEChERS method by adding 10 mL of ACN and stirring for 1 min. Then, 4 g of anhydrous MgSO_4_ and 1.5 g of NaCl were added and the mixture was vortexed for 1 min, followed by a centrifugation for 3 min at 7000 rpm. Four milliliters of the resulting supernatant were taken and diluted to 40 mL with water. After that, 40 mg of Fe_3_O_4_@PSA@COF was added to the extract and dispersed with vortex for 20 min. The magnetic sorbent was retained into the recipient with an external magnetic field and the supernatant was discarded. Next, the analytes were desorbed with 5 mL of ACN under sonication for 3 min. Finally, the ACN containing the analytes was recovered, dried with N_2_ at 40 °C, reconstituted in 400 µL of ACN and injected in the UHPLC-MS/MS system for analyte determination. The combination of the extraction step of the QuEChERS method and the m-dSPE with COF followed by UHPLC-MS/MS resulted in a very simple and efficient procedure that allowed obtaining recovery values in the range 75.9–103.0%, with LODs between 0.002 and 0.063 µg/kg.

**Figure 7:**
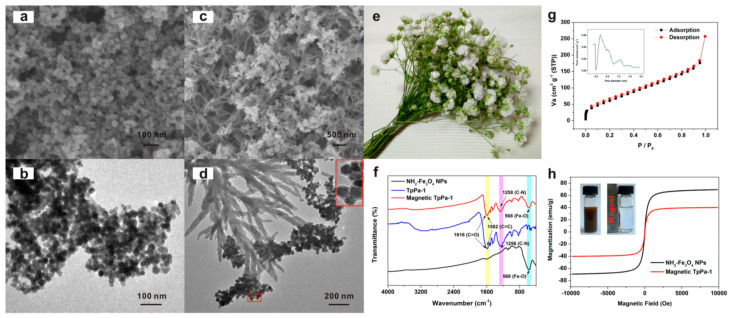
SEM images of the Fe_3_O_4_@NH_2_ (**a**) and Fe_3_O_4_@NH_2_@COF (**c**); TEM images of the Fe_3_O_4_@NH_2_ (**b**) and Fe_3_O_4_@NH_2_@COF (**d**); (**e**) photo of gypsophila bouquet; (**f**) FTIR spectra of the Fe_3_O_4_@NH_2_, COF and Fe_3_O_4_@NH_2_@COF; (**g**) nitrogen adsorption-desorption isotherm of the bouquet-shaped Fe_3_O_4_@NH_2_@COF, inset: pore-size distribution of this nanocomposites; (**h**) magnetization hysteresis loops of the Fe_3_O_4_@NH_2_ and Fe_3_O_4_@NH_2_@COF. Reprinted from [61] with permission of ACS Publications.

Following the trend previously mentioned, magnetic sorbents have been applied by dispersing them directly into the sample or sample extract depending on the sample pre-treatment applied before. Generally, low amounts of magnetic COFs have been necessary, always in the range of 5–100 mg, so all the procedures could be considered as magnetic-micro-dSPE (m-µ-dSPE). Sample or sample extract volumes between 0.5 and 200 mL were submitted to the extraction procedure, while adjusting their pH in order to improve the extraction performance when necessary [62,63,64,69,73,77,78,86,87,88]. Due to the high number of works in which magnetic COFs have been used as sorbents, a wide variety of mechanisms have been used to provide good dispersion of the sorbent into the sample matrix, including stirring [62,63], ultrasound [64,68,77,84], shaking [65,67,73,74,75,76,79,81,83,85,87], vortexing [66,69,71,72,78,82,88,89,90,91] and oscillation [86], although on certain occasions two different mechanisms have been combined to make the process more efficient [61,80]. As an example, He et al. [61] synthesized a Fe_3_O_4_@NH_2_@COF with a three-dimensional bouquet-like structure and applied it for the extraction of 6 PAHs from tap, lake and river water. To obtain this composite, amino-functionalized Fe_3_O_4_ NPs were firstly modified with Tp. Then, they were coated with the COF by adding a solution containing Tp and Pa building blocks under agitation at room temperature. After 30 min, the magnetic sorbent was isolated with an external magnetic field, washed and vacuum dried at 50 °C; they thereby obtained the desired bouquet-shape structure shown in Figure 7. The m-dSPE was made by dispersing only 5 mg of Fe_3_O_4_@NH_2_@COF in 200 mL of water sample previously filtered (0.22 µm), firstly under sonication for 1 min followed by 20 min of shaking. Then, the sorbent was magnetically isolated, the supernatant was discarded, and the analytes were desorbed with 12 mL of ACN (3 mL × 4) under ultrasound. All the eluates were collected and concentrated with N_2_ at 55 °C up to less than 1 mL and completed to 1 mL with ACN. Finally, 20 µL of the final extract was injected into the HPLC coupled to a fluorescence detection (FD) system for PAHs determination. This bouquet-shaped Fe_3_O_4_@NH_2_@COF showed an excellent extraction capacity and water stability, and its particular structure means that more porous COF is present in the final sorbent, contrary to classical single core-shell structure. Recovery values between 73% and 110% and LODs in the range 0.00024–0.00101 μg/L were obtained for PAHs, although this sorbent shows great potential to be used for the extraction of other compounds containing aromatic rings in their structures.

As a consequence of the wide variety of analytes and sorbents used for their extraction, an extensive number of solvents have been used to provide the best desorption of the analytes. Among them, ACN has clearly been the most commonly used, probably because of its intermediate polarity [61,62,63,66,71,72,75,79,80,81,82,90], although others such as MeOH [76,83,86,91], acetone [64,65,69], isopropanol [67], DCM [78] and EtOH [85], or even mixtures such as hexane/ethyl acetate [68], have also been used. In this sense, it is important to mention that in some cases, small amounts of bases (ammonium [70,73,74,84] or NaOH [77]) or acids (acetic acid [87] or formic acid [88,89]) have been added during the desorption step in order to improve the products.

Similarly to what it was discussed in the extraction step, desorption also requires a good dispersion of the sorbent into the desorption solvent in order to maximize the contact surface between both phases, which results in an enhancement of the procedure. In this sense, ultrasound has been the most popular dispersion mechanism [61,62,63,64,66,71,72,77,80,88,89,90], although vortexing [67,70,81,85,91], shaking [73,75,86,87], vibration [78] or even combinations of more than one mechanism [76,82] have also been applied.

Finally, and, as usual, after the application of every m-dSPE procedure, the analytes need to be properly separated and determined. Liquid chromatography, both HPLC [61,62,63,65,66,67,68,70,71,72,73,75,76,80,81,82,84,85,87,88,89,91] and UHPLC [74,77,79,83,90], has been the most extensively used, although some applications of GC [64,69,78,86] can also be found. These separation techniques have been coupled to different detection systems, including FD [61,63,72,76,79], UV [62,75,81,84,85,87], DAD [66,71,73,82,89], variable wavelength [80] and flame ionization detectors [64] (to MS [67,68,69,70,78] and MS/MS [65,74,77,83,86,88,90,91]). The combination of all these techniques with the good performances shown by the synthesized magnetic COFs has given rise to excellent sensitivity in all cases, with LODs in the low ppb level.

## 3. Solid-Phase Microextraction and Stir-Bar Sorptive Extraction

Current trends in sample preparation are focused on the development of new methodologies that meet the principles of green analytical chemistry [93]. For this reason, and despite the excellent extraction performance, simplicity and versatility shown by the already-mentioned conventional SPE, dSPE and m-dSPE, miniaturized sorbent-based techniques have emerged strongly in order to make the analytical procedures greener. In this sense, SPME constitutes without any doubt the perfect example of that, being one of the most extensively used sorbent-based extraction techniques, thanks to its simplicity, quickness and reduced volume of needed solvents which, in many cases, are absolutely unnecessary [94]. Since its introduction in 1990 [95], SPME has been subjected to a good number of modifications over the years leading to the introduction of different modalities, depending on analyte and sample characteristics. The introduction of COFs coatings, which was advantageous for the technique as a result of their modifiable pore size, high surface area, thermal stability and good selectivity, has clearly introduced a new and challenging SPME research area which is still in its infancy, like the rest of the sorbent-based extraction techniques in which COFs have been used. On the other hand, stir-bar sorptive extraction (SBSE), as a variant of SPME, followed a similar approach, incorporating COFs as viable and durable coatings for the extraction of a wide variety of analytes.

Table 3 summarizes the works already published dealing with the use of COFs as SPME and SBSE coatings. As can be seen in the table, COFs have been applied in SPME for the extraction and preconcentration of numerous analytes, such as PAEs [96,97], phenols [98], pesticides [99], PCBs [100], pyrethroids [101], chlorophenols (CPs) [98,102], benzene homologues [103], PAHs [104] and polybrominated diphenyl ethers (PBDEs) [105] from a wide variety of samples, including indoor air [103], environmental [105] and bottled water [96] samples, grilled meat [104], honey [98,102], canned peaches [102], fruits and vegetables [99,101], juices [97] and different aquatic organisms (snakeheads, catfish, bream, crucian, white shrimp and base shrimp) [100]. Concerning SBSE, the application of COFs as coatings has only been developed up to now for the extraction of PCBs from soils [106] and phenols from environmental water [107].

The main modality of SPME in which COFs have been employed has been head-space SPME (HS-SPME) [96,97,99,100,101,102,103], though there are also some direct immersion SPME (DI-SPME) applications [98,104,105]. Most COFs used under these modalities of SPME were prepared following a solvothermal synthesis approach [96,97,98,104,105], although other synthetic strategies have been successfully developed (also for SBSE coatings), involving ionothermal cyclotrimerization [107], solvent-free mechanochemical grinding [102], room temperature condensation [100] or the photo-induced thiol-ene click chemistry synthesis method [99], among others. Concerning fibers’ preparation, the most common approach has been the use of corroding agents to create an adequate rough surface on a stainless-steel wire prior to the incorporation of the coating. In general, aqua regia has been chosen as corroding agent in a large number of publications [96,97,99,100,101,103,104]. Nonetheless, other agents such as hydrofluoric acid [102,105] have also been viable options. Apart from the etching approach, a procedure in which a noble metal microstructured layer of silver is first deposited on top of the stainless-steel wire has also been proposed [98].

**Figure 8:**
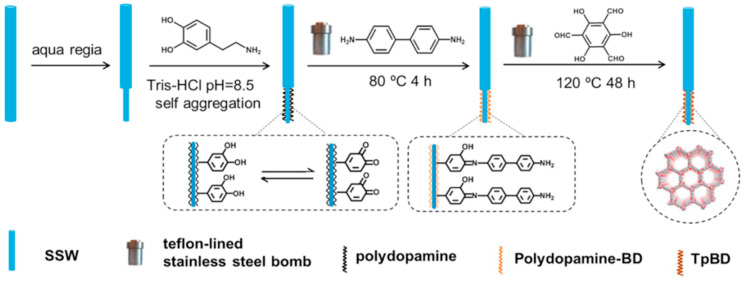
Fabrication of a Tp/BD COF bonded SPME fiber. Reprinted from [104] with the permission of Elsevier.

**Figure 9:**
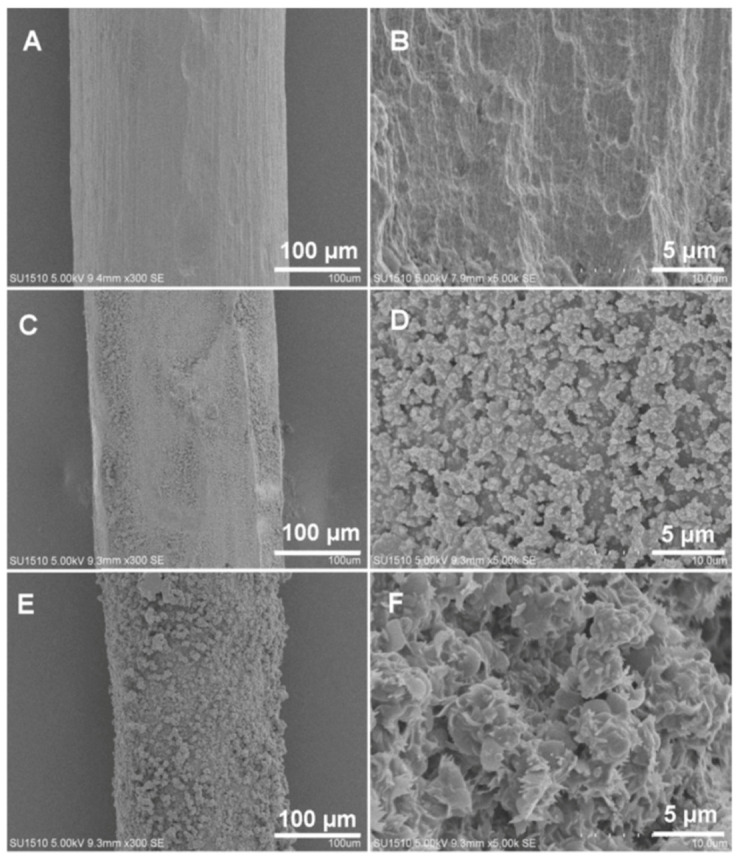
SEM images of an etched fiber (magnifications: 300× (**A**), 5000× (**B**)), a PDA-coated fiber (magnifications: 300× (**C**), 5000× (**D**)) and a Tp/BD COF bonded SPME fiber (magnifications: 300× (**E**), 5000× (**F**)). Reprinted from [104] with permission of Elsevier.

In many cases, the coating procedure involved the use of epoxy [96,97,103] or silicone [105] glues in order to retain COFs powder on the surface. Other cases first covered the wires with functionalized structures to retain the desired COFs: some procedures used PDA to obtain an initial coating and then incorporated APTES as the linker [99,101]. Other cases employed 3-mercaptopropyltriethoxysilane to prepare a silanol functionalized monolayer, and then, using a sol-gel solution of titanium butoxide and APTES, they incorporated the latter into the structure [98]. The use of APTES alone was also found to be feasible to generate the amino-functionalized surface to retain the COFs [100]. As an example, Figure 8 shows a schematic representation of the procedure followed by Ma et al. [104] for the fabrication of a COF (prepared from Tp and BD as building blocks) fiber. First, a stainless-steel wire is etched with aqua regia to obtain a rough surface. Then, dopamine polymerization (pH 8.5) is used to create an initial coating on the surface of the etched wire, and finally, the COF is synthesized and retained on the PDA coating through a solvothermal procedure. Figure 9 shows the SEM images obtained in each step, the differences being distinguishable between the etched fiber (Figure 9A,B), the PDA-coated fiber (Figure 9C,D) and the final product, the fiber with the bonded Tp/BD COF (Figure 9E,F). This coating was used for the extraction of 16 PAHs from grilled meat samples which were first homogenized using a blender. Then, homogenized meat was mixed with ACN and ultrasonicated. After centrifugation and collection of the supernatant the residual meat was subjected to another extraction. After evaporation of the collected extractions under a N_2_ stream, the residue was redissolved in 1 mL of ACN and diluted 1000 times with deionized water. Once the fiber was conditioned, the sample solution was subject to DI-SPME: 40 °C for 50 min under stirring (600 rpm). Finally, the fiber was inserted into a GC inlet for GC-MS/MS analysis. LODs and limits of quantification (LOQs) of the method were in the ranges of 0.02–1.66 and 0.07–5.52 ng/L, respectively. Recovery values ranged from 85.1% to 102.8% with RSD values lower than 8.4%.

Concerning coated stir-bars, one of the proposed procedures involved the use of a capillary glass with an iron wire within as the support structure for the polydimethylsiloxane (PDMS)/covalent triazine framework (CTF) coating, which was obtained using a sol-gel technique [107]. Another example used Fe_3_O_4_@mTiO_2_-COF as the coating and prepared the COFs via a condensation reaction with TAPB and TA and the m-NPs through a solvothermal approach. The magnetic properties of the proposed material were exploited to immobilize the coating on a magnetic stir-bar and perform a head-space sorptive extraction (HSSE) [106]. Said coating was applied to the extraction of seven PCBs from soils. For this purpose, soil samples were first dried at room temperature and crushed in a mortar to obtain a powder (about 200 meshes). Then, 10 mL of deionized water was mixed with 10 g of the soil sample and 10 µL of internal standard (difluorobiphenyl; 1 µg/mL) was added. After that, the coated stir-bar was hung in the headspace of a vial with the sample solution for the extraction at 50 °C for 30 min and 600 rpm. Next, the stir-bar was placed in a thermal desorption unit (splitless injection) and PCBs were cryo-focused. Finally, PCBs were subject to GC-MS analysis. The LODs and LOQs of the method were in the ranges of 0.003–0.006 and 0.013–0.020 µg/kg, respectively. Recovery percentages were in the range of 93.1–98.1% with RSD values lower than 4.6%.

The physical and chemical properties that determine the enrichment factors (EFs) of both techniques vary between the different COFs, there being tangible differences between compounds regarding the main effect driving the extraction. Nevertheless, it can be stated that all the selected COFs share common interactions with the target analytes, such as hydrophobic [97,98,99,100,101,102,103,105] or hydrophilic interactions [96], hydrogen bonding [96,101,107] and/or π–π stacking [96,97,98,99,100,101,102,103,104,105,107]. Other key properties to ensure a quantitative extraction were pore size [97,100,104,105], porosity [99,105] and surface areas [99,101,104]. For a better appreciation of these facts, Table 4 summarizes the main chemical and physical properties that, according to the authors, affect the EFs of the different analytes.

Regarding the recovery values obtained, and despite the fact that both SPME and SBSE are non-exhaustive extraction techniques, in many cases good recovery values were obtained (between 70.2% and 125.4%) with low RSD values (<12.7%) (see Table 3). Some examples are the work of Guo et al. [97], who analyzed 9 PAEs in juice samples using of 2,4,6-triphenoxy-1,3,5-triazine (TPT) COF fibers with recovery percentages between 79.4% and 110.3% and RSD values lower than 8.3% in HS-SPME, and the work of Zhang et al. [103], who also obtained good results in the analysis of 11 benzene homologues in indoor air using COF-SCU1 fibers with recovery values in the range 87.9–103.4% and low RSD values (<10.3%) in HS-SPME.

For a better characterization, coatings were subject to thermo-gravimetric analysis or other thermal stability studies, suggesting that all COFs were stable at temperatures above 290 °C; however, some COF coatings were able to withstand temperatures equal or higher than 400 °C [100,101,105,106,107] before thermal decomposition. Chemical stability was also evaluated by submerging coatings in different solvents (polar and non-polar) and acidic/basic solutions for a period of time. In general, the proposed COFs showed both good thermal and chemical stabilities.

Concerning the reproducibility and repeatability of the extractions, some differences were raised among the coatings (see Table 4). From the COFs evaluated, reproducibility results showed low RSD values; however, inter-batch studies were not always performed in the different materials in order to assure that the synthesis process proceeded as expected. For those cases where inter-batch reproducibility was studied, RSD values were acceptable with results below 12.1% [96,97,99,101,102,107].

In relation to reusability, in both SPME and SBSE it is important to reuse the fibers several times; otherwise, the effort to synthesize such microextractive coatings would be in vain. Data concerning the number of cycles in which each fiber/bar type has been used can also be found in Table 4. In general, all the fibers could be used above 100 adsorption/desorption cycles—an exception was the TPT-COF coating [97]. Concerning stir-bars’ reusability, such values were lower than those observed for other fibers (with the exception of OH-2,4,6-triphenoxy-1,3,5-benzene COF fiber) [96]. 

## 4. Conclusions

COFs have shown themselves to be promising sorbents due to their highly tunable properties via predictable control of structures and functionality. In conventional SPE, COFs have been broadly used based on the Tp building block, functionalized or not, but significant improvements in selectivity and sensitivity were achieved with hybrid COFs such as MICOFs and MOF@COFs. In m-dSPE, unlike in dSPE where few matrices were analyzed, mainly water samples, COFs have been applied for the extraction of diverse analytes from liquid, semi-solid and solid matrices. The combination of Tp and BD building blocks and Fe_3_O_4_ NPs has been the preferred choice in m-dSPE by in-situ growth of the COF around the m-NPs or previous functionalization of the latter with one of the building blocks or other functional groups. COFs have also been used successfully as fiber coatings in SPME, in both DI and HS modes, showing good thermal stability and reusability. Despite these all promising results, the synthesis of new functionalized or hybrid COFs and further development towards their practical use are still needed to expand their applications as superior sorbents for the selective extractions of target analytes, particularly hydrophilic compounds. In any case, there is no doubt that new applications will continue to appear in the near future.

## Figures and Tables

**Table 1:** Applications of COFs as sorbents in SPE.

Sorbent (COF Building Blocks)	Analytes	Matrixes	Separation and Detection Techniques	Extraction Conditions	Recovery (RSD)	LODs	Comments	Reference
COF (Azo and Tp)	4 benzoylurea insecticides	Juice, tomato and white radish	HPLC-VWD	-Sorbent amount: 25 mg -Conditioning: 5 mL acetone, 5 mL ACN and 5 mL water -Sample volume: 100 mL -Flow rate: 4.0 mL/min -Washing: 5 mL water:ACN 95:5 (*v*/*v*) -Sorbent drying: - (vacuum) -Desorption: 300 μL ACN	84.1–108.4% (3.4–6.2%)	0.10–0.20 μg/L for juice sample, and 0.05–0.10 μg/kg for tomato and white radish samples	This COF was found to be unstable in strong alkaline solutions.	[36]
COF (DA and Tp)	4 benzoylurea insecticides	Environmental water, fruit juice, fruits and vegetables	HPLC-UV	-Sorbent amount: 20 mg -Conditioning: 3 mL acetone, 3 mL ACN and 3 mL water -Sample volume: 100 mL -Flow rate: 2.0 mL/min -Washing: 5 mL ACN:water 1:10 (*v*/*v*) -Desorption: 200 μL ACN	85.5–112.7% (3.0–6.8%)	0.02–0.05 μg/L for water and juice samples, and 0.02–0.08 μg/kg for fruits and vegetables samples	-	[37]
MICOF (TAPB and Tp)	4 cyano pyrethroids	Vegetables, fruits and traditional Chinese medicines	HPLC-DAD	-Sorbent amount: 100 mg -Conditioning: 2 mL EtOH and 2 mL n-hexane -Sample volume: 2 mL -Flow rate: 7.5 mL/min -Washing: 1 mL n-butanol -Desorption: MeOH 4% HAc	94.3–102.7% (3.1–5.9%)	0.011–0.018 μg/kg	-	[38]
NH_2_@COF (TAPB and Dva. AIBN was added for the functionalization)	6 carboxylic acid pesticides	Ground water, tap water, river water and lake water	HPLC-DAD	-Sorbent amount: 100 mg -Conditioning: 5 mL NH_3_:MeOH 8:92 (*v*/*v*) and 5 mL water -Sample volume: 20 mL (pH 4) -Flow rate: 5.0 mL/min -Sorbent drying: 3 min (vacuum) -Desorption: 2 mL NH_3_:MeOH 8:92 (*v*/*v*) -Desorption flow rate: 3.0 mL/min	89.6–102.4% (0.03–7.10%)	0.01–0.06 μg/L	Four commercial sorbents (C_18_, phenyl-silica, silica and SAX) were compared obtaining better recovery values with NH_2_@COF.	[39]
COF (Tp and BD)	4 PEDs	Milk, carbonated and non-carbonated beverages	HPLC-UV	-Sorbent amount: 30 mg -Conditioning: 5 mL ACN 10% HAc and 5 mL water -Sample volume: 10 mL (pH 4) -Desorption: 4 mL ACN 10% HAc	82.0–96.3% (0.5–6.6%)	0.056-0.122 μg/L	-	[40]
NH_2_-MIL-68@COF (TFPA and TAPA. NH_2_-MIL-68-(CHO) was added to form the hybrid material)	6 SAs	Tap water, milk and pork	HPLC-VWD	-Sorbent amount: 8 mg -Conditioning: ACN and water -Sample volume: 2 mL (pH 7) -Flow rate: 0.2 mL/min -Desorption: 200 μL ACN -Desorption flow rate: 0.2 mL/min	68.9–103.8% (2.9–6.6%)	1–10 μg/L	A PT-SPE was carried out. This method is not suitable for rapid analysis with large sample volumes.	[41]
SNW-1@PAN nanofiber (MA and TA. PAN was added to synthesize the SNW-1@PAN electrospun nanofiber)	5 SAs	Pork and chicken	HPLC-DAD	-Sorbent amount: 12.5 mg -Conditioning: 1 mL MeOH and 1 mL water -Sample volume: 4 mL -Washing: 1 mL MeOH:water 1:9 (*v*/*v*) -Desorption: 1 mL MeOH 7.5% NH_3_	86.0–114.0% (1.6–9.3%)	1.7–2.7 μg/L	A PT-SPE was carried out.	[42]
COF (Tp and BD)	10 inorganic trace ions	Water and milk	ICP-MS	-Sorbent amount: 20 mg -Conditioning: HNO_3_ (0.5 M) and NH_4_Ac (0.1 M) -Sample volume: 20 mL (pH 5) -Flow rate: 1.5 mL/min -Desorption: 2 mL HNO_3_ (0.7 M)	81.0–96.0% (1.2–4.3%)	0.002–0.022 μg/L	On-line SPE was carried out. CTpBD was compared with TpBD, but TpBD only showed good recovery values for five of the target metal ions.	[43]
COF (Tp and Pa-NO_2_)	8 BAs	Meat	HPLC-FD	-Sorbent amount: 25 mg -Conditioning: 6 mL ACN and 6 mL water -Sample volume: 20 mL -Flow rate: 3.0 mL/min -Washing: 2 mL water:acetone 90:10 (*v*/*v*) -Sorbent drying: –(vacuum) -Desorption: 4 mL ACN	80.3–115.0% (6.6–12.0%)	4.6–12.9 μg/kg	Samples were derivatized with 40.0 μL of dansyl chloride solution in ACN before SPE.	[44]
COF (MA and Tp)	4 disinfection by-products	Drinking bottled water, tap water and pool water	GC-MS	-Sorbent amount: 100 mg -Conditioning: water and MeOH -Sample volume: 200 mL -Flow rate: 3.0 mL/min -Washing: 10 mL water -Sorbent drying: 3 min (vacuum) -Desorption: 16 mL DCM (8 mL × 2)	86.0–114.2% (0.5–6.3%)	0.0004–0.0063 μg/L	It was proved that this COF had a good chemical stability in different solvents.	[45]

ACN: acetonitrile; AIBN: azobisisobutyronitrile; Azo: 4,4′-azodianiline; BA: biogenic amine; BD: 4,4′-diaminobiphenyl; COF: covalent organic framework; DA: 2,6-diaminoanthraquinone; DAD: diode array detector; DCM: dichloromethane; Dva: 2,5-divinylterephthalaldehyde; EtOH: ethanol; FD: fluorescence detector; GC: gas chromatography; HAc: acetic acid; HPLC: high-performance liquid chromatography; ICP: inductively coupled plasma; LOD: limit of detection; MA: melamine; MeOH: methanol; MICOF: molecularly imprinted covalent organic framework; MS: mass spectrometry; Pa: *p*-phenylenediamine; PAN: polyacrylonitrile; Pa-NO_2_: 2-nitro-*p*-phenylenediamine; PED: phenolic endocrine disruptor; PT-SPE: pipette tip solid-phase extraction; RSD: relative standard deviation; SA: sulfonamide; SAX: strong anion exchange; SNW: Schiff base network; SPE: solid-phase extraction; TA: terephthaladehyde; TAPA: tris(4-aminophenyl)amine; TAPB: 1,3,5-tris(4-aminophenyl)benzene; TFPA: tris(4-formylphenyl)amine; Tp: 1,3,5-triformylphloroglucinol; UV: ultraviolet; VWD: variable wavelength detector.

**Table 2:** Applications of COFs as sorbents in dSPE and m-dSPE.

Sorbent (COF Building Blocks)	Analytes	Matrixes	Separation and Detection Techniques	Extraction Conditions	Recovery (RSD)	LODs	Comments	Reference
**dSPE**								
PS-DVB-GMA@COF (TFB and BD)	7 NSAIDs	Tap water, river water and hospital waste water	UHPLC-UV	-Sorbent amount: 20 mg -Sample volume: 10 mL (pH 4) -Adsorption time: 1 min (-) -Sorbent drying: plunger pulling/pushing -Desorption: 1.5 mL EtOH -Desorption time: 2 min (-)	84.3–99.6% (0.2–9.4%)	0.13–0.82 μg/L	An in-syringe dSPE was carried out. Adsorption and desorption steps were repeated thrice.	[54]
SiO_2_@MICOF (TFB and BD)	6 NSAIDs	River water and lake water	HPLC-UV	-Sorbent amount: 15 mg -Sample volume: 10 mL -Adsorption time: 5 min (US) -Desorption: 0.5 mL MeOH 1% NH_4_OH -Desorption time: 10 min (US)	77.3–111.6% (4.1–9.4%)	0.2–1.4 μg/L	A heterogeneous nucleation and growth synthesis method using ibuprofen as template was carried out.	[55]
COF (TFA and TAPB)	6 NACs	Lake water, waste water and tap water	HPLC-DAD	-Sorbent amount: 4 mg -Sample volume: 4 mL -Adsorption time: 5 min (US) -Sorbent drying: naturally at room temperature -Desorption: 4 mL ACN -Desorption time: 10 min (manual shaking)	84.0–112.3% (2.0–4.8%)	30–90 μg/L	Desorption process was repeated thrice. COF could be reused 9 times.	[56]
LZU1 (TFB and Pa)	6 fluorochemicals	Tap water, influent water, effluent water and metal plating waste water	SALDI-MS	-Sorbent amount: 0.06 mg -Sample volume: 2 mL -Adsorption time: 90 min (vibration) -Desorption: 30 µL MeOH:ACN 1:1 (*v*/*v*)	77.1–123.0% (–)	0.00004–0.017 μg/L	COF-LZU1 was used both as extraction sorbent and as SALDI-MS matrix. Once extraction was developed, the sorbent was isolated by centrifugation, and redispersed in a mixture MeOH:ACN 1:1 (*v*/*v*), and 1 μL of the dispersion was deposited for SALDI desorption.	[57]
PC-COF (Tp and Pa)	7 UV filters	Food packaging materials	HPLC-UV	-Sorbent amount: 20 mg -Extract volume: 100 mL -Adsorption time: 30 min (shaking) -Desorption: 1 mL ACN -Desorption time: 5 min (US)	86.4–96.7% (6.8–8.6%)	0.0012–0.0018 μg/kg	Positively charged COF was used. Food packaging materials were first put in contact with water at 70 °C for 2 h, and then water was analyzed.	[58]
Attapulgite@COF (Tp and Pa)	4 pyrethroids	River water	HPLC-DAD	-Sorbent amount: 10 mg -Sample volume: 8 mL -Adsorption time: 1 min (vortex) -Sample drying: N_2_ stream at 50 °C -Desorption: 1 mL ACN -Desorption time: 0.5 min (vortex)	71.2–88.7% (0.7–8.7%)	0.83–1.79 μg/L	The sorbent can be reused up to 5 times.	[59]
CSTF-COF (TFB and DATP)	8 *N*-nitrosamines	Bottled drinking water	UHPLC-MS/MS	-Sorbent amount: 20 mg -Sample volume: 40 mL (pH 5–7) -Adsorption time: 2 min (vortex) -Desorption: 5 mL MeOH -Desorption time: -(-)	88.6−105.5% (0.8–8.4%)	0.00013–0.00245 μg/L	The dSPE method showed to be simpler, faster, and more environmentally friendly than a conventional SPE one using HLB as sorbent. NDMA-d_6_ and NMOR-d_4_ were used as ISs.	[60]
**m-dSPE**								
Fe_3_O_4_@NH_2_@COF (Tp and Pa)	6 PAHs	Tap water, lake water and river water	HPLC-FD	-Sorbent amount: 5 mg -Sample volume: 200 mL -Adsorption time: 1 min (US) and 20 min (manual shaking) -Desorption: 12 mL ACN (3 mL × 4) -Desorption time: -(US)	73.0–110.0% (2–8%)	0.00024–0.00101 μg/L	The synthesis procedure allowed obtaining a bouquet-shaped magnetic COF with a large surface area and porosity.	[61]
Fe_3_O_4_@PEI@PDA@COF (BDBA)	Paclitaxel	Rat plasma	HPLC-UV	-Sorbent amount: 5 mg -Sample volume: 0.5 mL (diluted to 20 mL with a phosphate buffer solution at pH 6) -Adsorption time: 1 h (stirring) -Desorption: 200 µL ACN -Desorption time: 5 min (US)	99.4–103.7% (<2.3%)	0.02 μg/L	Plasma samples were firstly deproteinized with trichloroacetic acid. 7 PAHs were also extracted in order to evaluate the adsorption behaviour of the sorbent.	[62]
Fe_3_O_4_@PEI@LZU1 (TFB and Pa)	6 PAHs	Tap water, lake water, roadside soil and lakeshore soil	HPLC-FD	-Sorbent amount: 5 mg -Sample (extract for soils) volume: 20 mL of a phosphate buffer solution at pH 9 containing 1% ACN -Adsorption time: 30 min (stirring) -Desorption: 200 µL ACN -Desorption time: 3 min (US)	Water: 90.9–107.8% (2.6–4.1%) Soil: 85.1–105.0% (2.6–4.1%)	0.0002–0.020 μg/L	Soil samples were dried, grounded and extracted with ACN (US). After several processes, small volumes of ACN were diluted with buffer solution before m-dSPE.	[63]
Ni/CTF (DCB)	6 PAEs	Plastic bottles, a disposable plastic cup and boiling water previously contained in the plastic recipients	GC-FID	-Sorbent amount: 10 mg -Extract volume: 20 mL 3% NaCl (pH 7) -Adsorption time: 20 min (US) -Desorption: 150 µL acetone -Desorption time: 5 min (US)	Plastic materials: 85.8–119.0% (0.4–1.0%) Water: 83.2–113% (0.4–1.0%)	Plastic materials: 24–85 μg/kg Water: 0.15–0.53 μg/L	Plastic bottles or cups were firstly cut into small pieces and extracted with MeOH (US). The extract was adjusted to pH 7, NaCl was added and diluted with water. Boiling water was put in contact with plastic containers to let it cool down inside (about 1 h).	[64]
Fe_2_O_3_/CTF (DCB)	6 PFCs	Mineral water, river water, snow water and pond water	HPLC-MS/MS	-Sorbent amount: 50 mg -Sample volume: 25 mL -Adsorption time: 15 min (shaking) -Desorption: 2 × 3 mL acetone -Desorption time: 3 min each desorption (eddying)	81.8–114.0% (1.1–9.7%)	0.00062–0.00139 μg/L	-	[65]
Fe_3_O_4_@COF (Tp and BD)	15 PAHs	Smoked pork, wild fish, grilled fish, smoked bacon, coffee and river water	HPLC-DAD	-Sorbent amount: 5 mg -Extract volume (sample volume for water): 10 mL -Adsorption time: 12 min (vortex) -Desorption: 1 mL ACN -Desorption time: 15 min (US)	84.3−107.1% (2.5−4.3%)	0.00083–0.012 μg/L	Meat samples were firstly hydrolyzed, and PAHs were then extracted with ACN (US). A certain volume of the concentrated extract was diluted with water. Coffee samples were put in contact with hot pure water before extraction.	[66]
Fe_3_O_4_@COF (TAPB and TPA)	5 biphenols	Human serum	HPLC-MS	-Sorbent amount: 20 mg -Sample volume: 10 mL -Adsorption time: 10 min (shaking) -Desorption: 1.5 mL isopropanol (0.5 mL × 3) -Desorption time: 2 min each desorption (vortex)	93.0–107.8% (1.2–3.4%)	0.0010–0.078 μg/L	Serum samples were diluted 50-fold with water. BPA-d_16_ was used as IS.	[67]
Fe_3_O_4_@SiO_2_@NH_2_@COF-Aptamer (TMC and Pa)	Hydroxy-2′,3′,4′,5,5′-pentachlorobiphenyl	Human serum	HPLC-MS	-Sorbent amount: 30 mg -Sample volume: 40 mL -Adsorption time: 30 min (US) -Desorption: 400 µL hexane:ethyl acetate 1:1 (*v*/*v*) -Desorption time: -(-)	87.7–101.5% (–)	0.0021 μg/L	Human serum samples were diluted with a mixture of water:formic acid:2-propanol 50:40:10 (*v*/*v*/*v*) for protein denaturation and PCBs release. Sorbent selectivity was assessed using 3 more hydroxylated PCBs.	[68]
Fe_3_O_4_@PDA@COF (TFB and BD)	9 PAEs	Human plasma	GC-MS	-Sorbent amount: 20 mg -Sample volume: 3 mL (pH 7) -Adsorption time: 10 min (vortex) -Desorption: 500 µL acetone -Desorption time: 10 min (vibration)	90.5−98.7% (2.3–4.9%)	0.0025−0.01 μg/L	Human plasma proteins were firstly denaturated with HCl and trifluoroacetic acid.	[69]
Fe_3_O_4_@COF (TFB and BD)	4 estrogens and 3 stilbenes	Pregnant woman urine	HPLC-MS	-Sorbent amount: 20 mg -Sample volume: 20 mL -Adsorption time: 30 min (dispersion and incubation at room temperature) -Desorption: 0.5 mL ACN 0.01% NH_4_OH -Desorption time: 2 min (vortex)	80.6–111.6% (1.8-6.7%)	0.0002–0.0077 μg/L	Urine samples were diluted 20-fold with water. Deuterated estradiol was used as IS.	[70]
Fe_3_O_4_@COF (Tp and DA)	15 PAHs	Edible oil, grilled chicken and grilled fish	HPLC-DAD	-Sorbent amount: 10 mg -Extract volume: 10 mL -Adsorption time: 10 min (vortex) -Desorption: 1 mL ACN -Desorption time: 15 min (US)	85.5–104.2% (1.2–4.3%)	0.03–0.73 μg/L	Meat was hydrolyzed with KOH in water:EtOH 1:9 (*v*/*v*) and PAHs were extracted with ACN (US). Oil was diluted ACN:acetone 60:40 (*v*/*v*).	[71]
Fe_3_O_4_@COF (Tp and BD)	3 estrogens and 3 phenolic compounds	Chicken, shrimp and pork	HPLC-FD	-Sorbent amount: 10 mg -Extract volume: 10 mL -Adsorption time: 5 min (vortex) -Desorption: 1 mL ACN -Desorption time: 10 min (US)	89.6–108.9% (1.2–6.1%)	1.4–8.7 μg/L	Meat samples were firstly extracted with acetone (US).	[72]
Fe_3_O_4_@COF (TFPB and DATP)	6 FQs	Pork, milk and human plasma	HPLC-DAD	-Sorbent amount: 14 mg -Extract volume: 2 mL (pH 6) -Adsorption time: 60 min (shaking) -Desorption: 6 mL MeOH 1% NH_4_OH (2 mL × 3) -Desorption time: 20 min each desorption (shaking)	78.7–103.5% (2.9–6.2%)	0.25–0.5 μg/kg	Human plasma and pork were firstly extracted with ACN (vortex), while milk with trichloroacetic acid:MeOH 2:8 (*v*/*v*) (vortex).	[73]
Fe_3_O_4_@COF (BTCA and DETA)	19 dyes	Textile	UHPLC-MS/MS	-Sorbent amount: 100 mg -Extract volume: 50 mL -Adsorption time: 10 min (shaking) -Washing: 2 mL water and water 10% MeOH -Desorption: 1.5 mL MeOH 5% NH_4_OH (*v*/*v*) (0.5 mL × 3) -Desorption time: -(-)	72.2–107.0% (2.3–7.1%)	0.021–0.58 μg/kg	Textile samples were firstly cut into small pieces and extracted twice with MeOH (US) at 70 °C.	[74]
Fe_3_O_4_@NH_2_@COF (Tp and BD)	10 SAs	Pork, beef and chicken	HPLC-UV	-Sorbent amount: 20 mg -Extract volume: 20 mL -Adsorption time: 10 min (shaking) -Desorption: 5 mL ACN -Desorption time: 2 min (shaking)	82.0–94.0% (-)	0.28–1.45 μg/L	-	[75]
Fe_3_O_4_@COF (TAPB and TPA)	4 phenolic compounds	Tea drinks	HPLC-FD	-Sorbent amount: 40 mg -Sample volume: 25 mL -Adsorption time: 30 min (shaking) -Desorption: 3 mL MeOH -Desorption time: 3 min (US) and 3 min (vortex)	81.3–118.0% (0.1–8.3%)	0.08–0.21 μg/L	The selectivity of the developed sorbent was evaluated against other pollutants (phenols, PAHs, PCBs, 2,4-dichlorophenoxyacetic acid, perfluoroalkyl substances, and SAs), showing higher extraction efficiency for the target analytes.	[76]
Fe_3_O_4_@SiO_2_@COF (Tp and BD)	14 HAAs	Smokers and non-smokers urine	UHPLC-MS/MS	-Sorbent amount: 10 mg -Sample volume: 2 mL (pH 7) -Adsorption time: 1 min (US) -Washing: 2 mL water -Desorption: 4 mL ACN containing 300 µL 0.1% NaOH -Desorption time: 1 min (US)	95.4–129.3% (2.4–7.3%)	0.00014–0.00046 μg/L	Urine samples were firstly hydrolyzed with HCl at 70 °C. TriMeIQx, MeAαC-d_3_, AαC-15N_3_, Norharman-d_7_, and PhIP-d_3_ were used as ISs.	[77]
Fe_3_O_4_/G@PDA@COF (TFB and BD)	9 PAEs	Milk	GC-MS	-Sorbent amount: 20 mg -Sample volume: 10 mL (pH 7) -Adsorption time: 10 min (vortex) -Washing: with water × 3 -Desorption: 1.5 mL DCM (0.5 mL × 3) -Desorption time: 10 min each desorption (vibration)	91.4–105.2% (2.9–6.3%)	0.004–0.02 µg/L	Defatted milk samples were firstly deproteinized with HCl and trifluoroacetic acid.	[78]
Fe_3_O_4_@SiO_2_@COF (Tp and EB)	9 hydroxylated PAHs	Smokers and non-smokers urine	UHPLC-FD	-Sorbent amount: 10 mg -Sample volume: 5 mL -Adsorption time: –(shaking) and 1 min (incubation) -Desorption: 6 mL ACN (2 mL × 3) -Desorption time: -(-)	93.3–121.3% (0.5–3.5%)	0.0030–0.0096 µg/L	Urine samples were firstly hydrolyzed. Conventional SPE experiments were carried out in order to evaluate the accuracy of the m-dSPE method. The sorbent was preconditioned with 3 mL of water, 3 mL of MeOH and 3 mL of water before the m-dSPE.	[79]
Fe_3_O_4_@NH_2_@COF (TAP and BPDA)	6 SAs	Lake water, milk, pork, chicken and shrimp	HPLC-VWD	-Sorbent amount: 10 mg -Extract volume (sample volume for water): 40 mL -Adsorption time: 2 min (US) and 2 min (shaking) -Desorption: 400 µL ACN -Desorption time: -(US)	65.3–107.3% (3.2–6.7%)	0.2–1.0 µg/L	Milk samples were firstly deproteinized with HClO_4_. Meat samples were firstly extracted with ACN several times (US).	[80]
Fe_3_O_4_@SiO_2_@COF (Tp and DNBD)	6 nicotinoid insecticides	Cucumber and lettuce	HPLC-UV	-Sorbent amount: 10 mg -Extract volume: 50 mL -Adsorption time: 10 min (shaking) -Washing: 1 mL water -Desorption: 0.2 mL ACN (0.1 mL × 2) -Desorption time: 5 min each desorption (vortex)	77.5–110.2% (5.1–8.8%)	0.02–0.05 µg/L	Edible parts of vegetable samples were firstly blended and extracted with ACN thrice (shaking).	[81]
Fe_3_O_4_/PEG@SNW-1 (MA and TA)	5 benzoylurea pesticides	Tap water, industrial water and waste yard sewage	HPLC-DAD	-Sorbent amount: 20 mg -Sample volume: 8 mL -Adsorption time: 2 min (vortex) -Desorption: 1 mL ACN -Desorption time: 1 min (vortex) and 1 min (US)	64.0–107.2% (0.2–7.8%)	0.4–1.0 µg/L	-	[82]
CoFe_2_O_4_@CNT@COF (CTC and BDBA)	9 HAAs	Fried chicken and roast beef	UHPLC-MS/MS	-Sorbent amount: 15 mg -Extract volume: 10 mL -Adsorption time: 5 min (shaking) -Desorption: 4 mL MeOH -Desorption time: 5 min (–)	73.0–117.0% (1.3–9.1%)	0.0058–0.025 μg/kg	Meat samples were firstly cut into small pieces and digested with NH_4_OH:MeOH 7:3 (*v*/*v*) (US) thrice. The extracts were extracted with n-hexane several times.	[83]
Ni/CTF-SO_3_H (DCB)	2 benzimidazole fungicides	Fruits, vegetables, and juices	HPLC-UV	-Sorbent amount: 20 mg -Extract volume: 10 mL -Adsorption time: –(US) -Washing: 2 mL water and 2 mL MeOH -Desorption: 3 mL ACN:NH_4_OH 95:5 (*v*/*v*) -Desorption time: 5 min (-)	80.2–115.1% (4.9–11.5%)	1.23–7.05 µg/kg	Fruit and vegetable samples were firstly homogenized with water. Juice samples were directly treated. After pH adjustment to 10–11, solutions were extracted with ethyl acetate, evaporated and redissolved with 0.1 M HCl.	[84]
Fe_3_O_4_@SiO_2_@COF (Tp and BD)	5 benzimidazoles	Apple, lemon juice, grape juice and peach juice	HPLC-UV	-Sorbent amount: 20 mg -Sample (extract for apple) volume: 10 mL -Adsorption time: 20 min (shaking) -Desorption: 1.5 mL EtOH (0.5 mL × 3) -Desorption time: 2 min each desorption (vortex)	85.3–102.3% (2.1–8.6%)	2.5–2.9 µg/L	Apple samples were firstly blended. Apple and juice samples were 50-fold diluted before the m-dSPE procedure.	[85]
Fe_3_O_4_/COF (Tp and BD)	15 PAEs	Alcoholic carbonated beverage, milk beverage, beer, tea drink, milk tea, carbonated drinks, juice, and solid beverage	GC-MS/MS	-Sorbent amount: 30 mg -Sample volume: 30 mL (pH 7) -Adsorption time: 30 min (oscillation) -Washing: water -Desorption: 2 mL MeOH -Desorption time: 15 min (shaking)	79.3–121.8% (2.1–11.9%)	0.005–2.748 µg/L	Alcoholic carbonated beverage, beer and carbonated drink were degassed (US) before m-dSPE procedure.	[86]
Fe_3_O_4_@SiO_2_@NH_2_@COF@2-FPBA (Tp and DNBD)	5 MNTs	Human urine	HPLC-UV	-Sorbent amount: 10 mg -Sample volume: 0.95 mL (pH 7) -Adsorption time: 10 min (shaking) -Washing: 5 mL NaH_2_PO_4_ buffer (pH 7) and 5 mL water -Desorption: 1 mL 5% HAc -Desorption time: 30 min (shaking)	86.3–114.9% (2.8–14.4%)	0.31–0.54 µg/L	Blank urine samples were obtained by oxidizing the endogenous MNTs at 37 °C, and then Fe_3_O_4_@COF@2-FPBA NPs were used to extract endogenous MNTs from urine. Before m-dSPE, urine proteins were precipitated with ACN and the pH was adjusted to 7.	[87]
Fe_3_O_4_@COF@Au NPs@MPS (Tp and BD)	6 FQs	Pork, chicken and bovine	HPLC-MS/MS	-Sorbent amount: 10 mg -Extract volume: 10 mL (pH 5) -Adsorption time: 30 min (vortex) -Desorption: 1 mL formic acid:MeOH 4:6 (*v*/*v*) -Desorption time: 25 min (US)	82.0–110.2% (3.9–7.7%)	0.1–1.0 µg/kg	Meat samples were cut into small pieces and blended. Then they were digested with a mixture HCl:ACN 1:50 (*v*/*v*). Finally, they were extracted using ACN saturated n-hexane.	[88]
Fe_3_O_4_@COF (Tp and DA)	7 PGRs	Apple, orange, tomato, and cucumber	HPLC-DAD	-Sorbent amount: 15 mg -Extract volume: 10 mL -Adsorption time: 5 min (vortex) -Desorption: 1 mL ACN 1% formic acid -Desorption time: 10 min (US)	83.0–105.0% (0.7–4.5%)	4.68–7.51 µg/L	Fruit and vegetable samples were firstly cut into small pieces and homogenized, and then extracted with MeOH.	[89]
Fe_3_O_4_@PSA@COF (DHTA and TAPB)	20 OPPs	Watermelon, peach, and orange	UHPLC-MS/MS	-Sorbent amount: 40 mg -Extract volume: 40 mL -Adsorption time: 20 min (vortex) -Desorption: 5 mL ACN -Desorption time: 3 min (US)	75.9–103.0% (0.7–12.3%)	0.002–0.063 µg/kg	Grape was used as matrix for method optimization. Fruit samples were firstly homogenized and extracted using the first stage of the QuEChERS method (10 mg sample, 10 mL ACN, 1.5 g NaCl and 4 g anhydrous MgSO_4_). Final extract was diluted with H_2_O before m-dSPE.	[90]
Fe_3_O_4_@SiO_2_@NH_2_@COF (Tp and TFPDA)	6 PFCs	Milk	HPLC-MS/MS	-Sorbent amount: 20 mg -Sample volume: 20 mL -Adsorption time: 15 min (vortex) -Desorption: 1.5 mL MeOH -Desorption time: 15 min (vortex)	81.3–128.1% (0.02–9.70%)	0.000005–0.00005 μg/L	Milk samples were 1000-fold diluted. 13C^8^-PFOA was used as IS.	[91]

2-FPBA: 2-formylphenylboronic acid; ACN: acetonitrile; BD: 4,4′-diaminobiphenyl; BDBA: benzene-1,4-diboronic acid; BPDA: 4,4′-biphenyldicarboxaldehyde; BTCA: 1,3,5-benzenetricarboxaldehyde; CNT: carbon nanotube; COF: covalent-organic framework; CSTF: clover-shaped nano-titania functionalized; CTC: cyclotricatechylene; CTF: covalent triazine framework; DA: 2,6-diaminoanthraquinone; DAD: diode array detector; DATP: 4,4′-diamino-*p*-terphenyl; DCB: 1,4-dicyanobenzene; DCM: dichloromethane; DETA: diethylenetriamine; DHTA: 2,5-dihydroxyterephthalaldehyde; DNBD: 3,3′-dinitrobenzidine; dSPE: dispersive solid-phase extraction; DVB: divinyl benzene; EB: ethidium bromide; EtOH: ethanol; FD: fluorescence detector; FID: flame ionization detector; FQ: fluoroquinolone; G: graphene; GC: gas chromatography; GMA: glycidylmethacrylate; HAA: heterocyclic aromatic amine; HAc: acetic acid; HLB: hydrophilic-lipophilic balance; HPLC: high-performance liquid chromatography; IS: internal standard; LOD: limit of detection; LZU: Lan Zhou University; MA: melamine; m-dSPE: magnetic dispersive solid-phase extraction; MeOH: methanol; MNT: monoamine neurotransmitter; MPS: 3-mercaptopropanesulphonate; MS: mass spectrometry; MS/MS: tandem mass spectrometry; NAC: nitroaromatic compound; NDMA: *N*-nitrosodimethylamine; NMOR: *N*-nitrosomorpholine; NP: nanoparticle; NSAID: non-steroidal anti-inflammatory drug; OPP: organophosphorus pesticide; Pa: *p*-phenylenediamine; PAE: phthalic acid ester; PAH: polycyclic aromatic hydrocarbon; PC: positively charged; PCB: polychlorinated biphenyl; PDA: polydopamine; PEG: polyethylene glycol; PEI: polyethyleneimine; PFC: perfluorinated compound; PGR: plant growth regulator; PSA: *N*-[3-(trimethoxysilyl)propyl]ethylenediamine; PS: polystyrene; QuEChERS: quick, easy, cheap, effective, rugged, and safe; RSD: relative standard deviation; SA: sulphonamide; SALDI: surface-assisted laser desorption/ionization; SNW: Schiff base network; TA: terephthalaldehyde; TAP: tetraamino porphyrin; TAPB: 1,3,5-*tris*(4-aminophenyl)benzene; TFA: 2,3,5,6-tetrafluoroterephthalaldehyde; TFB: 1,3,5-triformylbenzene; TFPB: 1,3,5-*tris*(*p*-formylphenyl)benzene; TFPDA: 2,3,5,6-tetrafluoro-1,4-phenylenediamine; Tp: 1,3,5-triformylphloroglucinol; TPA: terephthaldicarboxaldehyde; UHPLC: ultra-high-performance liquid chromatography; US: ultrasound; UV: ultraviolet; VWD: variable wavelength detector; TMC: trimesoyl chloride.

**Table 3:** COFs’ applications as sorbents in SPME and SBSE.

Sorbent (COF Building Blocks)	Analytes	Matrixes	Separation and Detection Techniques	Extraction Conditions	Recovery (RSD)	LODs	Comments	Reference
**SPME**								
OH-TPB-COF (TPB-CHO and TH)	6 PAEs	Bottled water	GC-FID	HS mode: -Sample volume: –(ionic strength 20%, *w*/*v*). -Adsorption time/temperature: 50 min, 105 °C -Desorption time/temperature: 7 min, 250 °C	78.6–101.9% (1.2–7.2%)	0.032–0.451 µg/L	-	[96]
TPT-COF (TPT-CON_2_H_4_ and TA)	9 PAEs	Juice	GC-FID	HS mode: -Sample volume: 1 mL (ionic strength 20%, *w*/*v*) -Adsorption time/temperature: 40 min, 85 °C -Desorption time/temperature: 6 min, 250 °C	79.4–110.3% (0.6–8.3%)	0.01–0.31 µg/L	TPT-COF fiber was aged in the GC injection port at 250 °C for 30 min.	[97]
SNW-1 (TA and MA)	7 phenols	Honey	GC-MS	DI mode: -Sample volume: 20 mL (ionic strength 15%, *w*/*v*). -Adsorption time/temperature: 40 min, 25 °C (stirring) -Desorption time/temperature: 10 min, 280 °C	84.2–107.2% (3.8–12.7%)	0.04–0.50 µg/kg	Honey samples were dissolved in water with NaCl. Then, the solution was derivatized with BSTFA.	[98]
Cross-linked hydrazone COF (BTCH and HPA)	4 organochlorine pesticides	Cucumber	GC-ECD	HS mode: -Extract volume: 1 mL -Adsorption time/temperature: 40 min, 60 °C -Desorption time/temperature: 2 min, 250 °C	78.2–107.0% (1.2–8.3%)	0.0003–0.0023µg/kg	Cucumber samples were cut into pieces, homogenized and extracted with ACN (US).	[99]
COF (TFPB and BD)	7 PCBs	Snakeheads, catfish, bream, crucian, white shrimp and base shrimp	GC-MS/MS	HS mode: -Sample volume: 10 mL -Adsorption time/temperature: 50 min, 70 °C -Desorption time/temperature: 5 min, 300 °C	87.1–99.7% (–)	0.07–0.35 µg/L	Prior to the HS-SPME procedure, the fiber was conditioned at 310 °C for 30 min.	[100]
COF/PDA (BTCA and TH)	4 pyrethroid pesticides	Fruits and vegetables	GC-ECD	HS mode: -Extract volume: 1 mL -Adsorption time/temperature: 30 min, 50 °C -Desorption time/temperature: 2 min, 250 °C	75.6–106.3% (2.1–7.6%)	0.11–0.23 µg/kg	Fruit and vegetable samples were cut into pieces, homogenized and pyrethroids extracted with a n-hexane:acetone 1:1 (*v*/*v*) mixture (US). Prior to each extraction, the fiber was conditioned at 250 °C for 2 min.	[101]
COF (Tp and BD)	7 CPs	Honey and canned yellow peach	GC-MS	HS mode: -Sample volume: 12 mL (pH 11.0, ionic strength 25% *w*/*v*). -Adsorption time/temperature: 35 min, 40 °C (shaking and stirring) -Desorption time/temperature: 17 min, 250 °C	70.2–113.0% (4.8–11.9%)	0.3–1.8 µg/kg	Peach samples were homogenized (honey did not require pretreatment). Then, samples were dissolved in water with NaHCO_3_ and KCl (pH 11) and diluted. The solution obtained was derivatized with acetic anhydride adding TBP as IS. Prior to HS-SPME, fibers were conditioned at 280 °C for 2 h.	[102]
SCU1 (Pa and BTCC)	11 benzene homologues	Indoor air	GC-MS	HS mode: -Sample volume: 25 mL -Adsorption time/temperature: 20 min, 40 °C -Desorption time/temperature: 10 min, 250 °C	87.9–103.4% (3.4–10.3%)	0.00003–0.00015 µg/L	-	[103]
COF (Tp and BD)	16 PAHs	Grilled meat	GC-MS/MS	DI mode: -Solution volume: 1000 mL -Adsorption time/temperature: 50 min, 40 °C (stirring) -Desorption time/temperature: 4 min, 300 °C	85.1–102.8% (1.1–8.4%)	0.00002–0.00166 µg/L	Meat samples were homogenized and extracted twice with ACN (US). Prior to SPME procedure, the fiber was conditioned 310 °C until the baseline was stable.	[104]
TpPa-1 (Tp and Pa)	5 PBDEs	Ground water, drinking water, and pond water	GC-MS	DI mode: -Sample volume: 10 mL -Adsorption time/temperature: 40 min, 70 °C -Desorption time/temperature: 5 min, 300 °C	71.9–125.4% (2.3–8.7%)	0.0000058–0.000022 µg/L	Prior to analysis, the samples were filtered with 0.45 μm filter membranes. The TpPa-1 coating was conditioned at 280 °C for 12 h. Between SPMEs, the TpPa-1 coating was reconditioned at 280 °C for 5 min.	[105]
**SBSE**								
Fe_3_O_4_@mTiO_2_@COF (TAPB and TA)	7 PCBs	Soil	GC-MS	TD mode: -Sorbent amount: 50 mg -Extract volume: 10 mL -Adsorption time/temperature: 30 min, 50 °C (stirring) -Desorption: TDU in splitless mode -Cryofocusing	93.1–98.1% (1.5–4.6%)	0.003–0.006 µg/kg	Soil samples were dried at room temperature. Then, the sample was mixed with deionized water and difluorobiphenyl was added as IS. Cryofocusing was carried out in a CIS4 injector at a temperature of 20 °C using liquid CO_2_.	[106]
CTF-1 (TN and PDMS)	8 phenols	River water and lake water	HPLC-UV	LD mode: -Sorbent amount: 20 mg -Sample volume: 10 mL -Adsorption time/temperature: 50 min, - (stirring) -Desorption: 50 μL methanol:NaOH 10 mM 8:2 (*v*/*v*) (US) for 25 min	78.6–121.0% (0.02–7.40%)	0.08–0.30 µg/L	Water samples were filtered through 0.45 μm PTFE membrane. Stir bars were cleaned with MeOH (US) for 10 min.	[107]

BSTFA: *N*,*O*-Bis(trimethylsilyl)trifluoroacetamide; BTCA: 1,3,5-benzenetricarboxaldehyde; BTCC: benzene-1,3,5-tricarbonyl chloride; BTCH: 1,3,5-benzenetricarbohydrazide; BD: 4,4′-diaminobiphenyl; CP: chlorophenol; CTF: covalent triazine framework; DI: direct immersion; ECD: electron capture detector; FID: flame ionization detector; GC: gas chromatography; HS: head space; HPA: 4-hydroxyisophthalaldehyde; IS: internal standard; LD: liquid desorption; LOD: limit of detection; MA: melamine; MS: mass spectrometry; MS/MS: tandem mass spectrometry; Pa: *p*-phenylenediamine; PAE: phthalic acid ester; PAH: polycyclic aromatic hydrocarbon; PBDE: polybrominated diphenyl ether; PCB: polychlorinated biphenyl; PDMS: polydimethylsiloxane; PTFE: polytetrafluoroethylene; RSD: relative standard deviation; SBSE: stir-bar sorptive extraction; SNW: Schiff base network; SPME: solid phase microextraction; TA: terephthalaldehyde; TAPB: 1,3,5-*tris*(4-aminophenyl)benzene; TBP: 2,4,6-tribromophenol; TD: thermal desorption; TDU: thermal desorption unit; TFPB: 1,3,5-*tris*(p-formylphenyl)benzene; TH: terephthalohydrazide; TN: terephthalonitrile; Tp: 1,3,5-triformylphloroglucinol; TPB: 2,4,6-triphenoxy-1,3,5-benzene; TPT: 2,4,6-triphenoxy-1,3,5-triazine; UHPLC: ultra-high-performance liquid chromatography; US: ultrasound; UV: ultraviolet.

**Table 4:** Main chemical and physical properties influencing the extraction efficiency of COFs used in SPME and SBSE.

COF	Analytes	Main Chemical Additionally, Physical Properties								Reference
		π-π Stacking	Hydrogen Bonding	Hydrophilic Interactions	Hydrophobic Interactions	Host-guest Interactions	Pore Size	High Porosity	Large Surface Area	
OH-TPB COF	PAEs	X	X	X						[96]
TPT COF	PAEs	X			X		X			[97]
SNW-1	Phenols	X			X					[98]
Cross-linked hydrazone COFs	Pesticides				X			X	X	[99]
TFPB-BD	PCBs	X			X		X			[100]
PDA COF	Pyrethroids	X	X		X				X	[101]
TpBD COF	CPs	X			X					[102]
COF-SCU1	Gaseous benzene homologues	X			X					[103]
TpBD	PAHs	X					X		X	[104]
TpPa1	PBDEs	X			X		X	X		[105]
Fe_3_O_4_@mTiO_2_-COF	PCBs						X			[106]
CTF-1	Phenols	X	X		X					[107]
